# Evaluation of efficacy and mechanism of *Bacillus velezensis* CB13 for controlling peanut stem rot caused by *Sclerotium rolfsii*

**DOI:** 10.3389/fmicb.2023.1111965

**Published:** 2023-02-16

**Authors:** Shu Jia, Ce Song, Hai Dong, Xujie Yang, Xinghai Li, Mingshan Ji, Jin Chu

**Affiliations:** ^1^College of Plant Protection, Shenyang Agricultural University, Shenyang, China; ^2^Sericultural Research Institute of Liaoning Province, Fengcheng, China; ^3^Institute of Plant Protection, Liaoning Academy of Agricultural Sciences, Shenyang, China

**Keywords:** *Bacillus velezensis* CB13, *Sclerotium rolfsii*, peanut stem rot, defense enzyme, colonization, soil rhizosphere microbiome, control efficiency

## Abstract

Peanut stem rot, caused by *Sclerotium rolfsii*, considerably affects crop productivity. Application of chemical fungicides harms the environment and induces drug resistance. Biological agents are valid and eco-friendly alternatives to chemical fungicides. *Bacillus* spp. are important biocontrol agents that are now widely used against several plant diseases. This study aimed to evaluate the efficacy and mechanism of a potential biocontrol agent *Bacillus* sp. for controlling peanut stem rot caused by *S. rolfsii*. Here, we isolated a strain of *Bacillus* from pig biogas slurry that considerably inhibits the radial growth of *S. rolfsii*. The strain CB13 was identified as *Bacillus velezensis* on the basis of morphological, physiological, biochemical characteristics and phylogenetic trees based on the 16S rDNA and *gyrA*, *gyrB*, and *rpoB* gene sequences. The biocontrol efficacy of CB13 was evaluated on the basis of colonization ability, induction of defense enzyme activity, and soil microbial diversity. The control efficiencies of *B. velezensis* CB13-impregnated seeds in four pot experiments were 65.44, 73.33, 85.13, and 94.92%. Root colonization was confirmed through green fluorescent protein (GFP)-tagging experiments. The CB13-GFP strain was detected in peanut root and rhizosphere soil, at 10^4^ and 10^8^ CFU/g, respectively, after 50 days. Furthermore, *B. velezensis* CB13 enhanced the defense response against *S. rolfsii* infection by inducing defense enzyme activity. MiSeq sequencing revealed a shift in the rhizosphere bacterial and fungal communities in peanuts treated with *B. velezensis* CB13. Specifically, the treatment enhanced disease resistance by increasing the diversity of soil bacterial communities in peanut roots, increasing the abundance of beneficial communities, and promoting soil fertility. Additionally, real-time quantitative polymerase chain reaction results showed that *B. velezensis* CB13 stably colonized or increased the content of *Bacillus* spp. in the soil and effectively inhibited *S. rolfsii* proliferation in soil. These findings indicate that *B. velezensis* CB13 is a promising agent for the biocontrol of peanut stem rot.

## 1. Introduction

Peanuts (*Arachis hypogaea* L.), an important economic and oilseed crop worldwide, address nutritional needs in developing countries owing to their protein content, taste, and affordability (Toomer, 2018; Höfer et al., 2022). Regular peanut consumption has been linked to low risk of developing heart disease (Bouchenak and Lamri-Senhadji, 2013), hypertension (Jones et al., 2014), and cancer (Li et al., 2022). However, peanuts are susceptible to many diseases, of which the peanut stem rot caused by *Sclerotium rolfsii* (teleomorph: *Athelia rolfsii*; Yan et al., 2021) is the most damaging, as it reduces peanut yields and causes large-scale economic losses. In China, the stem rot affects 10–50% of the peanut crop, with yield losses ranging from 50 to 100% (Chen K. R. et al., 2018). In central Vietnam, 5–25% of peanut plants are infected by *S. rolfsii* (Yan et al., 2021). *S. rolfsii* is very difficult to control, as it has more than 500 plant hosts and produces sclerotia that overwinter in soil and remain viable for a long period even in the absence of a susceptible host (Le et al., 2018; Yu et al., 2022).

Peanut stem rot is mainly controlled using agricultural measures, such as adjusted row patterns (Sconyers et al., 2007), crop rotation, deep ploughing (Timper et al., 2001), use of resistant varieties (Luo et al., 2020), and application of chemicals (Standish et al., 2019). However, despite achieving some control, each method also has limitations. When arable land resources are limited, it is difficult to promote and apply crop rotation, and the lack of resistant varieties with high yield and quality renders it difficult to meet production demand. Therefore, production remains dominated by the use of chemical control measures. However, long-term use of chemicals not only pollutes the environment, causes pesticide residue accumulation, and leads to drug resistance in pathogens (Zhou et al., 2022) but also adversely affects soil biodiversity (Scheeomaker and Kassteele, 2014). Therefore, an efficient and environment-friendly control method is needed to control peanut stem rot.

Biological control is a valid and eco-friendly alternative to chemical fungicides (Djordje et al., 2018). *Trichoderma* spp. (Motlagh et al., 2022), *Streptomyces* spp. (Jacob et al., 2018), *Pseudomonas* spp. (Liu et al., 2022), and *Bacillus* spp. (Yang et al., 2017; Li, 2018; Chen et al., 2020a) were all found to exhibit antagonistic activities against *S. rolfsii* and reduce stem rot incidence and severity in pot experiments. Among biocontrol strains, *Bacillus* spp. are considered to be the best candidate probiotic strains because of their broad-spectrum antagonistic activities against plant pathogens (Djordje et al., 2018), growth-promoting effect, and the ability to increase plant yields (Saxena et al., 2020; Lu et al., 2021; Pan et al., 2022). *Bacillus* strains exhibit biocontrol capacity predominantly through the inhibition of pathogen growth, induction of systemic resistance in plants, and competition for ecological niches (Djordje et al., 2018). This study aimed to identify new strains for the biological control of peanut stem rot and provide a scientific basis for the development and application of biocontrol agents. To this end, we isolated a typical *Bacillus* sp. from pig biogas slurry that inhibits the radial growth of *S. rolfsii*. Furthermore, we evaluated the competence of one strain, CB13, as a potential biocontrol agent through its colonization on peanut root and rhizosphere soil, defense enzyme activity of peanut, and the efficacy of peanut stem rot. In addition, microbial biocontrol agents, if used as a seed coating or directly added to the soil, could affect the indigenous microbes in soil (Wang et al., 2020). Studying the impact of CB13 on the rhizosphere microbiome will help in comprehensively evaluating the biocontrol potential of this strain.

## 2. Materials and methods

### 2.1. Strains and variety

The biocontrol strains, isolated from pig biogas slurry, were obtained from the Institute of Plant Protection, Liaoning Academy of Agricultural Sciences. *S. rolfsii* was provided by the College of Plant Protection, Shenyang Agricultural University. PHT0-P43GFPmut3a, a plasmid carrying chloramphenicol-and green fluorescent protein-encoding genes, was provided by the Rice Disease Research Laboratory, Institute of Plant Protection, Liaoning Academy of Agricultural Sciences. A peanut rot susceptible variety of the Fuyu four red peanut (Fan, 2021) was used for the analysis (Registration Number: GPD [2018]220156).

### 2.2. Influence of antagonistic bacteria on radial growth of *Sclerotium rolfsii*

Plate confrontation method (Shan et al., 2013) was employed to determine the inhibitory effect of antagonistic bacteria against *S. rolfsii*. The *S. rolfsii* isolates were grown on a potato dextrose agar (PDA) medium at 35°C for 5 days. Approximately 5-mm disks of *S. rolfsii* were placed at the center of a 90-mm diameter PDA plate, and the bacteria were inoculated 25 mm away from the center. In the controls, 5-mm disks of *S. rolfsii* were placed at the center of a 90-mm diameter PDA plate. The plates were cultured at 28°C until the fungus in the control plate covered the full plate. A light microscope was used to examine the morphology of the hyphae present at the edge of the colony. Antagonistic activity of endophytes was evaluated by the inhibition rate (IR), which was calculated using the following formula:


$$IR\left(\%\right)=\frac{\left(D-5\right)-\left(d-5\right)}{D-5}\times 100$$

where *D* is the diameter of *S. rolfsii* disks in control treatment, *d* is the diameter of *S. rolfsii* with the antagonistic bacteria, and 5 is the diameter of the disks inoculated with *S. rolfsii*. The strain showing the strongest antagonistic activity against *S. rolfsii* was selected for further studies.

### 2.3. Transmission electron microscopy

The edges of *S. rolfsii* mycelia cultured with the antagonistic bacteria were fixed in 3% glutaraldehyde in 0.1 M sodium cacodylate buffer (pH 7.4) at 4°C for 24 h. The samples were treated as described by Jia et al. (2020). The samples were observed using a transmission electron microscope (Hitachi H-7650; Hitachi Ltd., Tokyo, Japan) at 115 kV.

### 2.4. Identification of the biocontrol strain CB13

The biocontrol strain CB13 was observed with an optical microscope. The physiological and biochemical tests, including Gram staining, methyl red test, starch hydrolysis, peptone hydrolysis, gelatin liquefaction test, VP test, catalase test, and salt tolerance test, were performed with reference to previous literature (Zhu, 2011). The CB13 strain was identified using 16S rDNA, *gyrA*, *gyrB*, and *rpoB* gene sequences. The genomic DNA of CB13 was extracted using a bacterial genomic DNA FastPrep Extraction Kit (Sangon Biotech, Shanghai, China). DNA was quantified using a NanoDrop ND100 spectrophotometer (Thermo Fisher Scientific, Waltham, MA, United States) and stored at −20°C. Partial sequences of 16S rDNA, *gyrA*, *gyrB*, and *rpoB* were amplified using specific primers (Supplementary Table S1; Ki et al., 2009; Cao et al., 2014; Jing et al., 2019). The 50 μL polymerase chain reaction (PCR)-system contained 5 μL of 10× PCR buffer, 2 μL dNTPs, 1 μL Pfu DNA polymerase (Takara Bio Inc., Beijing, China), 3 μL genomic DNA template (25 ng/μL DNA), 2 μL of each primers (0.1 μM), and 35 μL deionized distilled water. The PCR program included a denaturation step at 94°C for 5 min, followed by 30 cycles of denaturation at 95°C for 1 min, annealing at 50–63°C for 30 s, and elongation at 72\u00B0C for 1 min, followed by a final elongation cycle at 72°C for 5 min, after which the PCR product was kept at 4°C. The PCR products were purified using a DNA gel extraction kit (Qiagen, Hilden North Rhine-Westphalia, Germany) and sent to Sangon Biotech (China) for sequencing. DNA sequence homology analysis was performed using the BLAST tool of the GenBank nucleotide database of the National Center for Biotechnology Information (Bethesda, MD, United States).^1^ Multiple alignments with sequences of closely related *Bacillus* strains and sequence similarity calculations were performed using CLUSTAL X (Thompson et al., 1997). Phylogenetic trees were constructed with Mega 6.0 (Kim et al., 1993) using the neighbor-joining method with 1,000 bootstrap resampling replications (Felsenstein, 1985).

### 2.5. Pot assays for stem rot disease control

A bacterial suspension of the CB13 bacterial inoculum was prepared at a concentration of 10^7^ CFU/mL. The bacteria were seeded at a density of 1% into 100 mL lysogeny broth (LB) liquid medium and shaken at 150 rpm at 35°C for 24 h to obtain a bacterial inoculum for plant treatment.

The phytopathogen inoculum was prepared by autoclaving wet sorghum seeds in a 150 mL Erlenmeyer flask three times and then infecting them with mycelium plugs of *S. rolfsii* (6 mm diameter). The Erlenmeyer flask was maintained at 28°C until abundant mycelial growth was observed (approximately 7–10 days).

The pot experiment was conducted four times, in June and August 2021 and 2022. The peanut seeds were surface disinfected as described by Lu et al. (2015). The surface-sterilized seeds were sown in plastic pots filled with nutrient soil, with each pot containing 4–5 seeds. Three peanut plants were maintained after seedling growth (at temperature 28 ± 5°C and relative humidity 65 ± 5%) for 30 days. The peanut seedlings were divided into four treatment groups, with 20 pots in each group, and the experiments were performed in triplicate. Treatment groups were as follows: (A) CK—infected peanut plants without treatment; (B) THI—infected peanut plants sprayed with 20 mL of 240 g/L thifluzamide (diluted 500×; Zhejiang Hangzhou Yulong Chemical Co. LTD; thifluzamide treatment group); (C) RI—infected peanut plants watered with 20 mL bacterial inoculum (diluted 10×); (D) ST—seeds were soaked in bacterial inoculum (diluted 10×) for 24 h before sowing. The plants were treated thrice with CB13 and chemical agents once every 7 days. Infection with *S. rolfsii* was performed 1 day after the third treatment. Approximately 3 g of infected sorghum grains was used to inoculate the root of the plant, and the plants were examined after 5 days. The extent of the stem rot disease was scored as 0 for no stem lesion, 1 for small stem lesion, and 2, 3, and 4 for the wilting and death of ≤25, 26% ~ 50, and > 50% of whole plants in each group, respectively. The disease incidence rate (DIR), disease index (DI), and control efficacy (CE) were calculated as follows (Li et al., 2017):


DIR=nN×100;DI=∑(Ni×i)N×4×100;CE(%)=DICK−DITDICK×100

where *n* is the number of infected plants, *N* is the total number of investigated plants, Ni is the number of infected plants at a certain severity level, i is certain severity level, DI_CK_ is the disease index in control, and DI_T_ is the disease index in treatment.

### 2.6. Colonization of green fluorescent protein-tagged CB13 in peanut roots and rhizosphere soil

The natural transformation method (Li, 2011) was used to obtain GFP-labeled strains. Peanut seedlings were watered with 10 mL of CB13-GFP strain seed liquid at a concentration of 1 × 10^7^ CFU/mL, and flower roots and root soil were taken on the 3rd, 6th, 11th, 16th, 23rd, 30th, 40th, and 50th days after treatment. The roots in large granular soil were carefully removed, placed in a bottle containing 50 mL sterile water, and oscillated for 30 min. The soil suspension was shaken; 20 mL of the soil diluent was placed in a bottle, and the dry weight of soil in the soil suspension was determined; the residual soil was diluted to form a slurry gradient, and a coated chloramphenicol-resistant plate count was determined. The roots were dried with absorbent paper, weighed, cut into small segments of approximately 0.5 mm, ground fully with sterile water, diluted in gradient, and coated with a chloramphenicol screening medium (10 μg/mL concentration). The number of CB13-GFP colonies was determined after 2 days.

### 2.7. Influence of CB13 on the activities of defense enzymes in peanut plants

The seed disinfection and potted plant-handling methods were conducted as described above. After 30 days of seedling growth (at a temperature of 28 ± 5°C and relative humidity of 65 ± 5%), one plant per pot was maintained for inoculation. There were four treatments, as follows, with 20 pots in each treatment: (A) Uninoculated—uninfected and untreated plants; (B) Uninfected CB13—seeds soaked in bacterial inoculum (diluted 10×) for 24 h before sowing; (C) Inoculated—plants infected with *S. rolfsii* alone; (D) CB13 + infection—seeds soaked in bacterial inoculum (diluted 10×) for 24 h before sowing and then infected with *S. rolfsii*. Leaves from the same part of the plant were sampled at five time points (0, 12, 24, 48, and 72 h) post-infection, immediately wrapped in a tin foil, and frozen in liquid nitrogen. Enzyme activity was measured according to the kit manufacturer’s protocols (Sangon Biotech (Shanghai) Co., LTD, Shanghai, China).

### 2.8. Analysis of peanut rhizosphere microbiome

Seed disinfection and potted plant-handling were performed as described above. Peanut seedlings were divided into four treatments, with three pots per treatment, repeated in triplicate. Treatment groups were as follows: (A) untreated peanut plants (without pathogen or bioagent), labeled as “NS group;” (B) peanut plants watered with 20 mL of bacterial inoculum alone, labeled as “CS group;” (C) peanut plants infected with *S. rolfsii* alone, labeled as “SS group;” (D) peanut plants watered with 20 mL of bacterial inoculum and then infected with *S. rolfsii* 24 h later, labeled as “CSS group.” CB13 treatment was performed once every 7 days a total of three times; infection with *S. rolfsii* was performed 1 day after the third treatment, and soil samples were collected 10 days later. The root soils of the peanut plants were collected according to Courchesne’s root-shaking method (Courchesne and Gobran, 1997), and three soils were mixed together to form one soil sample. Microbial DNA was extracted from soil samples using the E.Z.N.A.^®^ soil DNA Kit (Omega Bio-Tek, Norcross, GA, United States) according to the manufacturer’s protocols. The final DNA concentration and purification were determined using a NanoDrop 2000 UV–vis spectrophotometer (Thermo Scientific, Wilmington, DE, United States), and DNA quality was checked using 1% agarose gel electrophoresis. The internal transcribed spacer (ITS) regions were amplified with primers ITSI and ITS2R, and V3-V4 hypervariable regions of the bacterial 16S rDNA gene were amplified with primers 338F and 806R (Supplementary Table S1; Xu et al., 2016) using a thermocycler PCR system (GeneAmp 9,700, ABI; Thermo Fisher Scientific, Waltham, MA, United States). PCR reactions were conducted using the following program: 3 min of denaturation at 95°C; 35 cycles (16S rDNA; 27 cycles) of denaturation at 95°C for 30 s, annealing at 55°C for 30 s, and elongation at 72°C for 45 s; and a final extension at 72°C for 10 min. PCR was performed in triplicate using 20 μL reaction mixtures containing 2 μL (16S rDNA, 4 μL) of 5× FastPfu Buffer, 2 μL of 2.5 mM dNTPs, 0.8 μL of each primer (5 μM), 0.4 μL FastPfu Polymerase, and 10 ng template DNA. The resulting PCR products were extracted from a 2% agarose gel, further purified using the AxyPrep DNA Gel Extraction Kit (Axygen Biosciences, Union City, CA, United States), and quantified using QuantiFluor™-ST (Promega, Madison, WI, United States).

### 2.9. Preprocessing of sequence data

Raw FASTQ files were de-multiplexed using an in-house perl script, and then quality-filtered by fastp (version 0.19.6; Chen S. F. et al., 2018) and merged by FLASH (version 1.2.7; Tanja and Steven, 2011) with the following criteria: (i) the 300 bp reads were truncated at any site receiving an average quality score of <20 over a 50 bp sliding window, and the truncated reads shorter than 50 bp were discarded, reads containing ambiguous characters were also discarded; (ii) only overlapping sequences longer than 10 bp were assembled according to their overlapped sequence. The maximum mismatch ratio of overlap region is 0.2. Reads that could not be assembled were discarded; (iii) Samples were distinguished according to the barcode and primers, and the sequence direction was adjusted, exact barcode matching, 2 nucleotide mismatch in primer matching. Then, the optimized sequences were clustered into operational taxonomic units (OTUs) using UPARSE (version 7.1; Edgar, 2013) with 97% sequence similarity level. The most abundant sequence for each OTU was selected as a representative sequence. The taxonomy of each 16S rDNA was analyzed by RDP Classifier (version 2.11)^2^ against the Silva (version 138)^3^ 16S rDNA database using confidence threshold of 70% (Cole et al., 2009). The taxonomy of each ITS gene sequence was analyzed by RDP Classifier against the UNITE (version 8.0)^4^ using confidence threshold of 70% (Cole et al., 2009).

### 2.10. Quantitation of *bacillus* spp. and *Sclerotium rolfsii* in peanut rhizosphere soil

To determine the content in peanut rhizosphere soil, the 16S rDNA*/*ITS copies of *Bacillus* spp./*S. rolfsii* were determined using real-time quantitative PCR (qPCR) by designing specific primers for *Bacillus* spp.: B1F/B1R and *S. rolfsii*: SRITSF/SRITSR (Supplementary Table S1; Pravi et al., 2014). The qPCR was performed in a 20-μL reaction mixture that consisted of 2 μL of template DNA, 10 μL of 2 × SYBR Mix, 0.25 μL of each primer, and 7.5 μL of ddH_2_O. The PCR protocol included a 5-min initial denaturation at 95°C, 40 cycles of 95°C for 30 s, 56°C for 30 s, and 72°C for 40 s. A standard curve that shows the relationship between copy numbers logarithm of *Bacillus* spp./*S. rolfsii* and Ct values was constructed, as described by Jing et al. (2019). The copies of *Bacillus* spp. and *S. rolfsii* were determined by using LineGene 9600 Plus (FQD-96A) Fluorescence Quantitative Thermal Cycler (Bioer Technology, China). The content was expressed by log copies DNA·g^−1^ soil.

### 2.11. Statistical analysis

Data were expressed as mean ± SD, and a suite of statistical analysis (SPSS 19 and MS Excel 2007) was used to find the mean and standard error. Statistical differences between treatments were analyzed using one-way ANOVA test at a 5% significance level. The software Mothur (Schloss et al., 2009) was used to calculate the alpha diversity index under random sampling, and alpha diversity and richness were determined at the OTU level using the Shannon index and Chao1 index, respectively. The Wilcoxon rank-sum test was used to compare statistical differences between groups. Principal coordinates analysis (PCoA) was conducted based on a Bray–Curtis dissimilarity matrix computed from the samples. The one-way analysis of similarity (ANOSIM) test (Clarke and Warwick, 2001) with 999 permutations was conducted to determine statistical significance. The functions of bacteria and fungi in peanut rhizosphere soil were analyzed using PICRUSt2 (version 2.2.0).^5^ The *p* value was corrected using a multiple test with false discovery rate (Fdr) and results with *p* < 0.05 were considered statistically significant. Differential abundance analysis for sequence count data was performed using DESeq2 (version 1.14.1; Love et al., 2014), with significance defined as *p* < 0.05, and Benjamini-Hochberg-adjusted *p* < 0.05. Unless otherwise stated, statistical analyses and plotting were carried out using R software (version 3.3.1).

## 3. Results

### 3.1. Influence of antagonistic bacteria on radial growth of *Sclerotium rolfsii*

**Figure 1:**
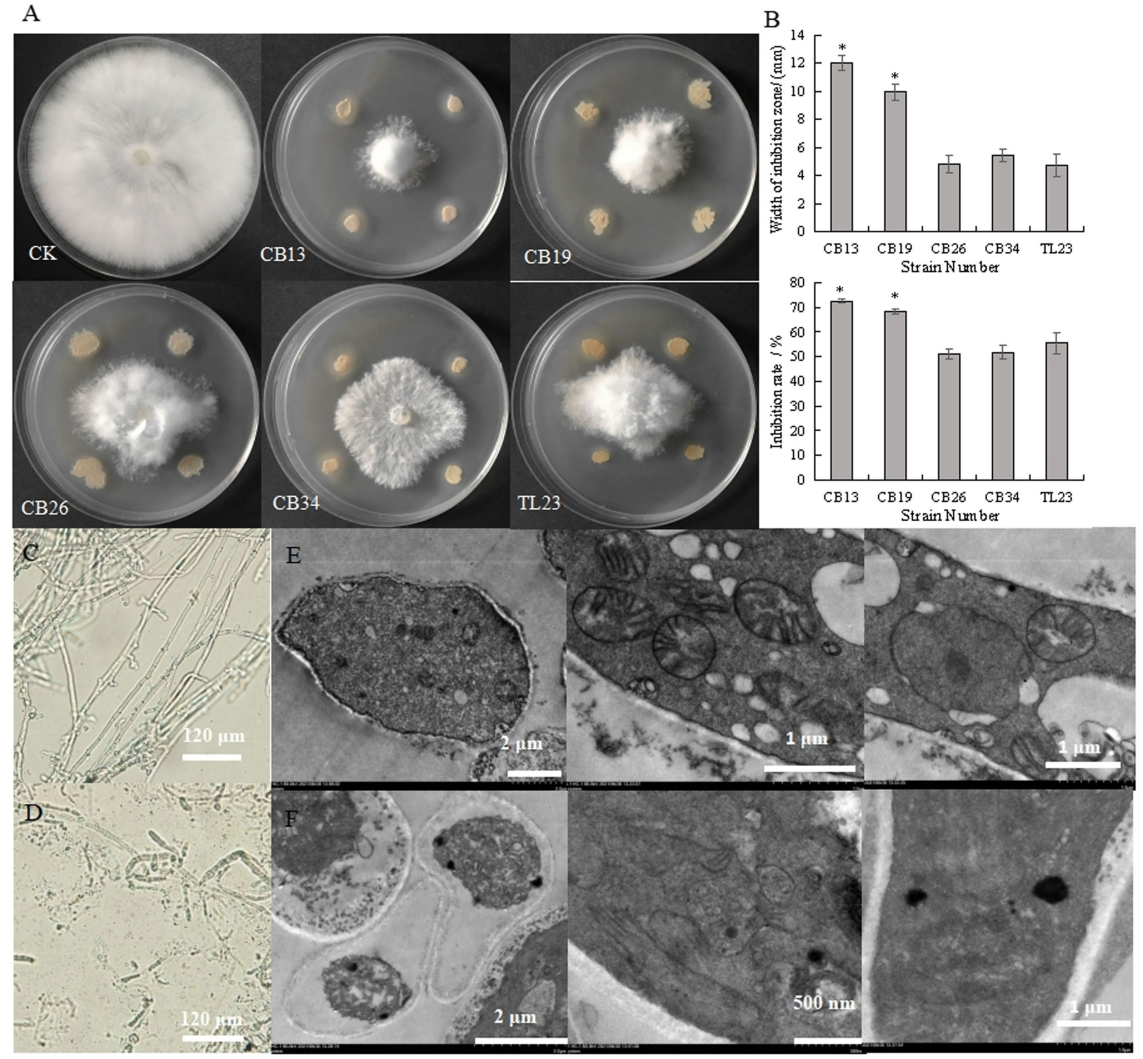
Inhibitory effect of antagonistic bacteria on the radial growth and mycelium cells of *Sclerotium rolfsii*. **(A)** Inhibitory effect of antagonistic bacteria on the mycelia growth of *S. rolfsii*; **(B)** width of inhibition zone and inhibition rate of antagonistic bacteria against *S. rolfsii*; **(C)** The structure of normal *S. rolfsii* mycelium observed *via* light microscopy; **(D)** The structure of *S. rolfsii* mycelium cultured with *Bacillus velezensis* CB13 observed *via* light microscopy. **(E)** The structure of normal *S. rolfsii* mycelium cells, mitochondria, and cell nuclei observed *via* transmission electron microscopy; **(F)** The structure of *S. rolfsii* mycelium cells, mitochondria, and cell nuclei cultured with *B. velezensis* CB13 observed *via* transmission electron microscopy. Data are the means ± SE of three repeated experiments. Significant differences are shown using asterisks (*p < 0.05, **p < 0.01, ***p < 0.001). Mycelia were cultured at 28°C for 5 days.

The antagonistic bacteria effectively inhibited the radial growth of *S. rolfsii* (Figures 1A,B). The CB13 strain exhibited the highest inhibition zone and IR against the radial growth of *S. rolfsii*, at 12.00 mm and 72.56%, respectively. Thus, the CB13 strain was selected for further studies.

Under the light microscope, the hyphae of *S. rolfsii* with normal growth were smooth and uniform (Figure 1C), while some hyphae of the inhibited *S. rolfsii* appeared darker, ablated, and ruptured, with vesicles present at the top (Figure 1D). Based on the transmission electron micrographs, normal *S. rolfsii* mycelial cells appeared full, with intact mitochondria, cell and nuclear membranes, and gathered nucleoli (Figure 1E), whereas the mycelial cells of *S. rolfsii* co-cultured with CB13 appeared pyknotic, with disintegrated mitochondria, cleaved nuclear membranes, and separated nucleoli (Figure 1F).

### 3.2. Identification of the biocontrol strain CB13

**Figure 2:**
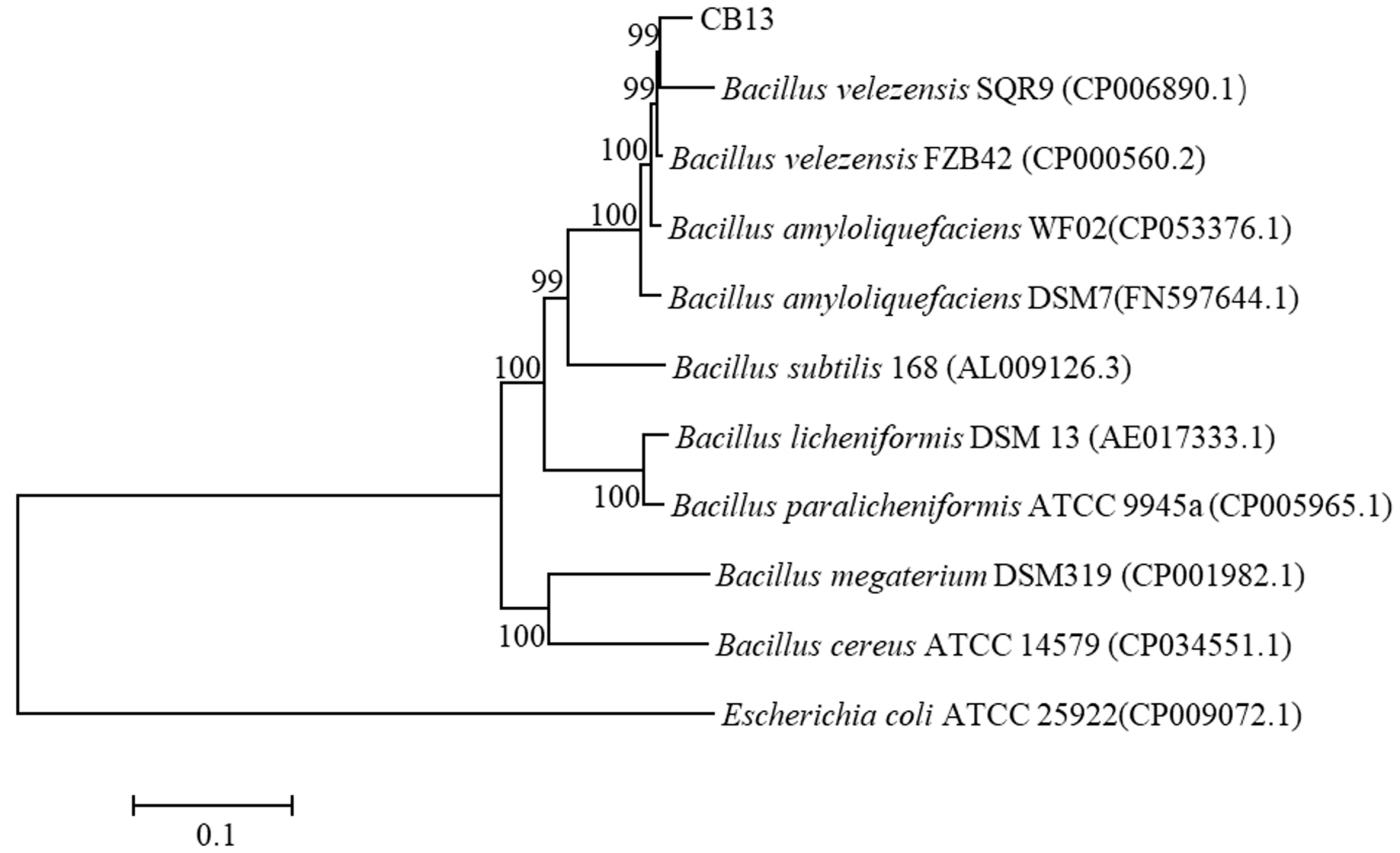
Phylogenetic trees based on the 16S rDNA, *gyrA* gene, *gyrB* gene, and *rpoB* gene sequences. The phylogenetic trees were constructed with the neighbor-joining method using the software package Mega, version 6.0, after multiple sequence alignments using Clustal X. GenBank accession numbers of bacterial strains are shown in parentheses. The number at each branch is the percentage of times the group of strains in that branch occurred, based on 1,000 cycles, *via* bootstrap analysis.

On LB medium, the colonies of CB13 appeared nearly round and milky-white–opaque, with dry folds on the surface and irregular edges. Optical microscopy identified the CB13 strain as rod-shaped with a size of 0.5 μm × 1.5–3.5 μm. The results of physiological and biochemical tests performed on strain CB13 are shown in Supplementary Table S2. Based on these results, along with morphological examinations, strain CB13 was preliminarily classified as a strain of the *Bacillus* spp. Multigene 16S rDNA, *gyrA*, *gyrB*, and *rpoB* phylogenetic analysis revealed 99% consistency between strain CB13 and *Bacillus velezensis* (Figure 2). Accordingly, strain CB13 was identified as *B. velezensis* and named *B. velezensis* CB13 based on the multigene sequence analysis and morphological, physiological, and biochemical characteristics.

### 3.3. Pot assays for stem rot disease control

**Figure 3:**
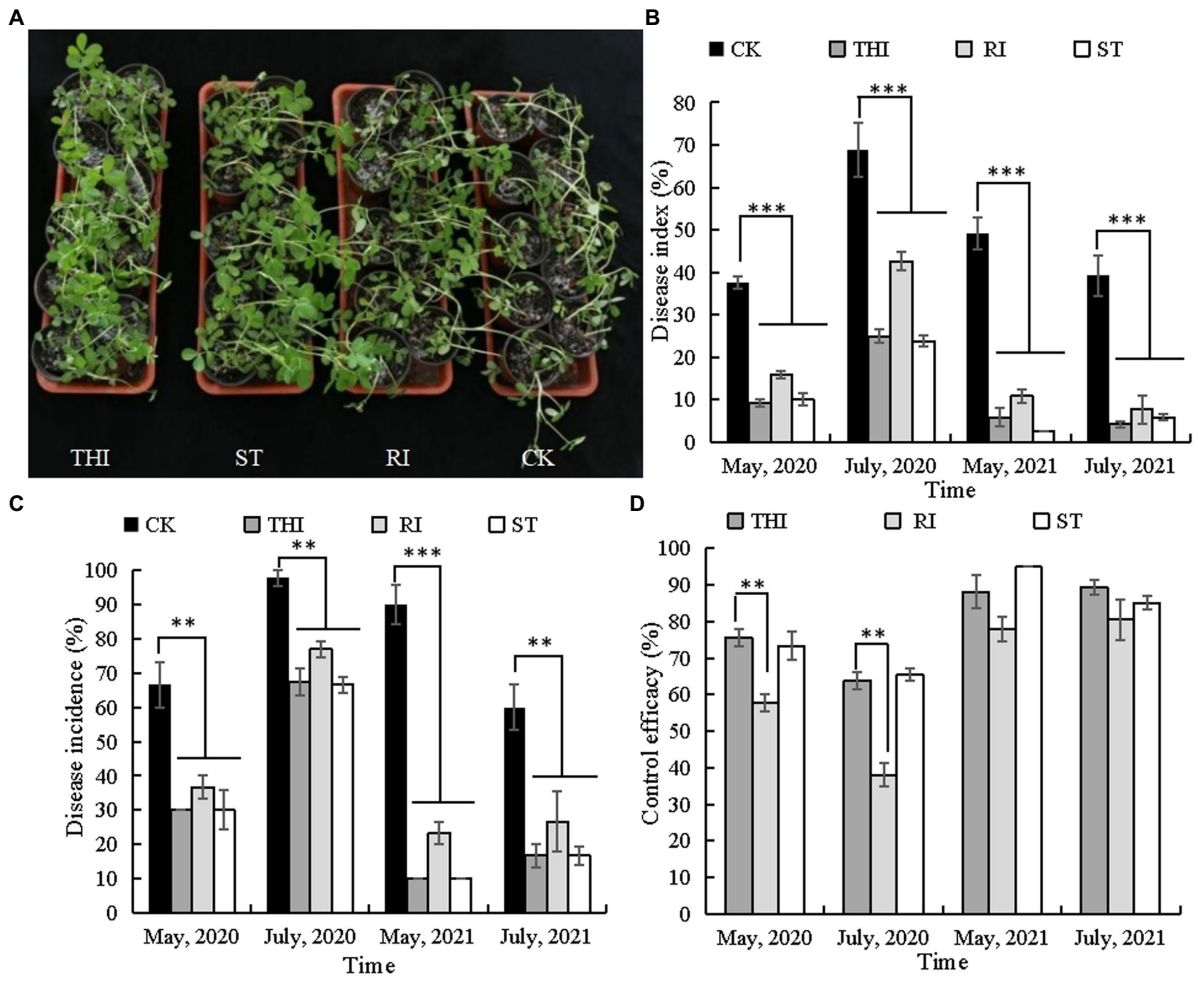
*Bacillus velezensis* CB13 reduced the disease incidence and disease index of peanut stem rot in pot assays. **(A)** Pot assays for stem rot disease control; **(B)** disease index; **(C)** disease incidence; **(D)** control effect. CK: infected peanut plants without treatment; THI: infected peanut plants sprayed with 20 mL of 240 g/L thifluzamide (diluted 500×); RI: infected peanut plants watered with 20 mL of bacterial inoculum (diluted 10×); ST: seeds were soaked in bacterial inoculum (diluted 10×) for 24 h before sowing. Data are the means ± SE of three repeated experiments. Significant differences are shown using asterisks (*p < 0.05, **p < 0.01, and ***p < 0.001).

*Bacillus velezensis* CB13 treatment reduced the incidence and disease index of peanut stem rot in pot assays (Figure 3). Thifluzamide, RI, and ST significantly reduced stem rot incidence and disease index (*p* < 0.05). There were no significant differences in stem rot incidence and disease index between the chemical control and ST (*p* > 0.05); however, the disease index (May 2020 and July and May 2021) and disease incidence (May 2021) were significantly higher than those in RI (*p* < 0.05). The control efficiencies of ST in four pot experiments were 65.44, 73.33, 85.13, and 94.92%. The levels of control effect achieved with thifluzamide or ST had no significant differences (*p* > 0.05) but were significantly higher than those achieved with RI (*p* < 0.05).

### 3.4. Colonization of green fluorescent protein-tagged CB13 in peanut roots and rhizosphere soil

**Figure 4:**
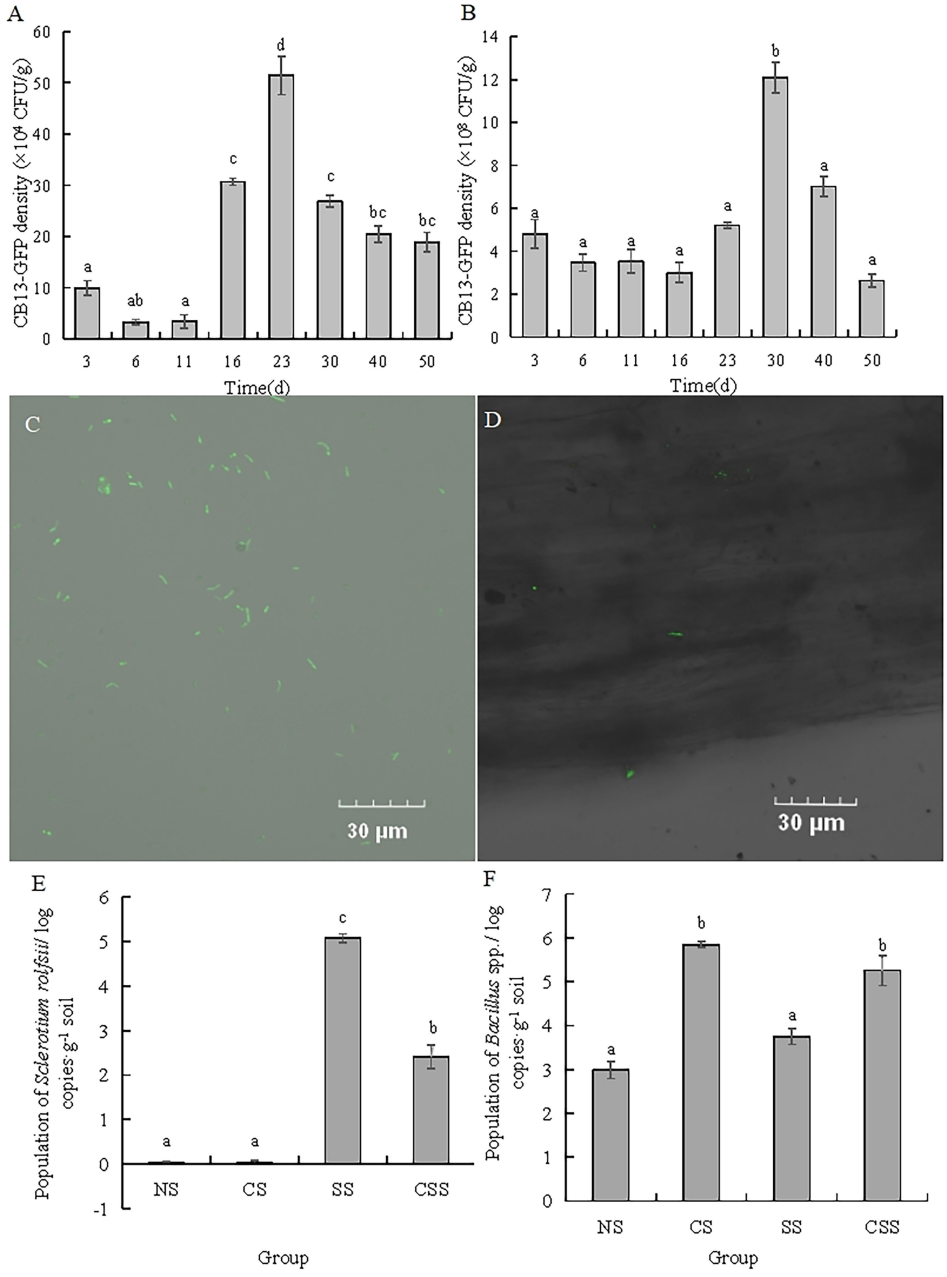
Analysis of colonization of *Bacillus velezensis* CB13 and *Sclerotium rolfsii* in peanut roots and rhizosphere soil. **(A)** The density of CB13-green fluorescent protein (GFP) in peanut root; **(B)** The density of CB13-GFP in peanut rhizosphere soil; **(C)** Fluorescence of bacterial cells emitted by strain CB13-GFP under the fluorescence microscope; **(D)** The colonization of CB13-GFP strain in root under the fluorescence microscope; **(E)**The quantification of *Bacillus* spp. in peanut rhizosphere soil by real-time quantitative PCR; **(F)** The quantification of *S. rolfsii* in peanut rhizosphere soil by qPCR; NS: uninoculated soil; CS: Soil inoculated with *B. velezensis* CB13; SS: Soil inoculated with *S. rolfsii*; CSS: Soil inoculated with *B. velezensis* CB13 and *S. rolfsii*. Data are the means ± SE of three repeated experiments. Different letters above error bars indicate significant differences according to one-way ANOVA test (*p* < 0.05).

The antagonistic strain *B. velezensis* CB13 was labeled with GFP using a natural transformation method (Figure 4C). The ability of *B. velezensis* CB13 to colonize peanut roots and rhizosphere soil was evaluated over 50 days (Figures 4A,B). CB13-GFP reached its highest density in peanut root after 23 days (51.44 × 10^4^ CFU/g of peanut root), which decreased thereafter to a final density of 18.89 × 10^4^ CFU/g of peanut root after 50 days. Furthermore, CB13-GFP reached its highest density in rhizosphere soil after 30 days (12.10 × 10^8^ CFU/g of rhizosphere soil), which decreased thereafter to a final density of 2.62 × 10^8^ CFU/g of rhizosphere soil after 50 days. The root colonization of CB13-GFP was observed under a fluorescence microscope (Figure 4D).

### 3.5. Influence of *Bacillus velezensis* CB13 on the activities of defense enzymes in peanut plants

**Figure 5:**
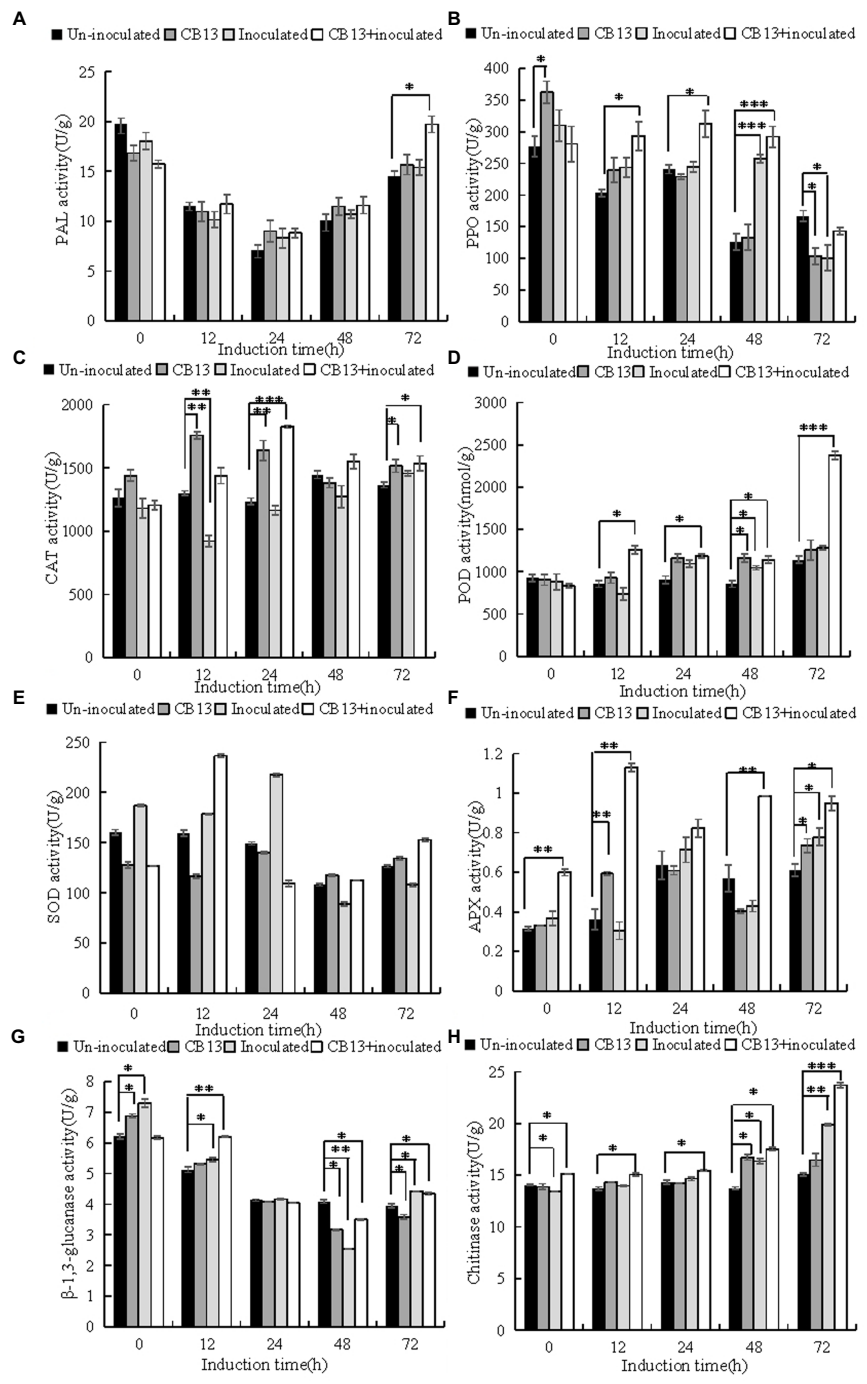
Effects of *Bacillus velezensis* CB13 treatment on the activities of enzymes related to resistance in *Sclerotium rolfsii*-induced peanut leaves. **(A)** phenylalanine ammonia-lyase; **(B)** polyphenol oxidase; **(C)** peroxidase; **(D)** catalase; **(E)** superoxide dismutase; **(F)** ascorbate peroxidase; **(G)** β-1,3-glucanase and **(H)** chitinase. Uninoculated, uninfected, and untreated plants; CB13, peanut plants watered with 20 mL of bacterial inoculum alone; Inoculated peanut plants infected with *S. rolfsii* alone; CB13 + inoculated, seeds were soaked in bacterial inoculum for 24 h before sowing and then infected with *S. rolfsii*. Data are the means ± SE of three repeated experiments. Significant differences are shown using asterisks (*p < 0.05, **p < 0.01, and ***p < 0.001).

The activities of defense enzymes increased to varying degrees in infected leaves after *B. velezensis* CB13 treatment compared to those in infected leaves without treatment (Figure 5). At 12 h, the activities of superoxide dismutase (SOD) and β-1,3-glucanase (GLU) in the CB13 + inoculated group increased significantly by 1.32 and 1.14 folds, respectively, compared to those in the inoculated group, and then decreased. The activities of polyphenol oxidase (PPO), ascorbate peroxidase (APX), catalase (CAT), phenylalanine ammonia-lyase (PAL), peroxidase (POD), and chitinase (CHI) in the CB13 + inoculated group increased significantly and were higher than those in the positive control group at all time points examined after inoculation. The activities of POD, CAT, and APX increased significantly by 1.72, 1.57, and 3.72 folds, respectively, compared to those in the inoculated group.

### 3.6. Analysis of peanut rhizosphere microbiome

After the removal of low-quality reads, 420,323 valid bacterial reads and 785,669 valid fungal reads were obtained from 12 samples. The number of valid bacterial reads ranged from 26,649 to 44,340 among the 12 samples, and the number of valid fungal reads ranged from 57,323 to 83,454. The sequences representing OTUs were identified with 97% similarity (Supplementary Tables S3, S4). The rarefaction curves for individual samples were close to the platform period, indicating that sequencing depth reflected the microflora richness (Supplementary Figure S1).

**Figure 6:**
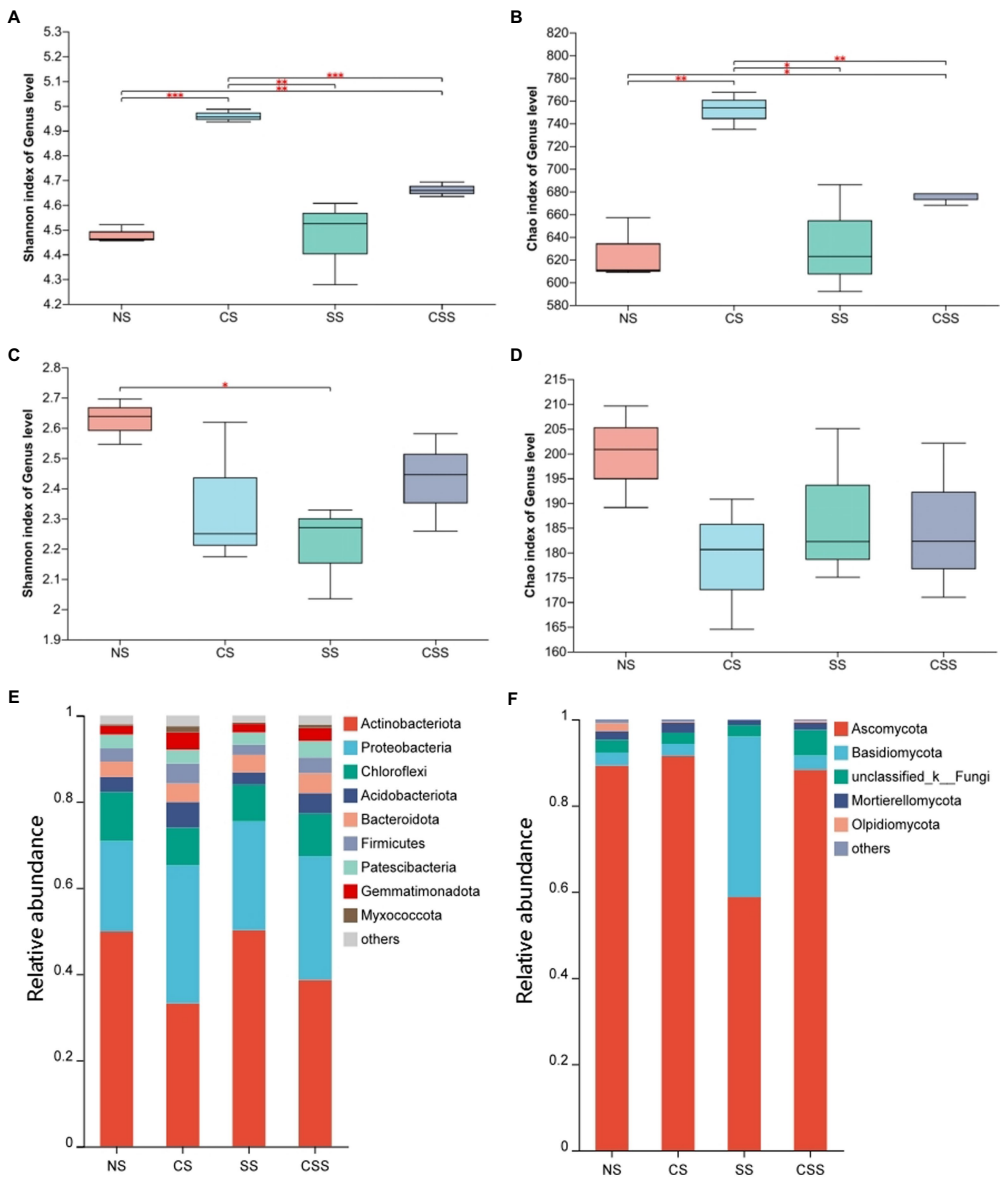
The diversity and composition of peanut rhizosphere microbiome. **(A)** Boxplot of soil bacterial diversity based on Shannon indices on the genus level; **(B)** Boxplot of soil bacterial richness based on Chao1 indices on the genus level; **(C)** Boxplot of soil Fungal diversity based on Shannon indices on the genus level; **(D)** Boxplot of soil Fungal richness based on Chao1 indices on the genus level; Differences between the means in each group were compared using a Wilcoxon rank-sum test (*p < 0.05, **p < 0.01, and ***p < 0.001). The top and bottom whiskers indicate the maximum and minimum values, respectively, and the hyphen represents the median value; **(E)** Bacterial composition of soil samples at the phylum level. **(F)** Fungal composition of soil samples at the phylum level. NS, uninoculated soil; CS, Soil inoculated with *Bacillus velezensis* CB13; SS, Soil inoculated with *Sclerotium rolfsii*; CSS, Soil inoculated with *B. velezensis* CB13 and *S. rolfsii*.

The Shannon index for the soil microflora revealed that bacterial diversity in the four treatment groups decreased in the order CS > CSS > SS > NS, while fungal diversity decreased in the order NS > CSS > SS > CS; these results were consistent with the Simpson’s index values (Figure 6). The Shannon index of species diversity indicated that the bacterial diversity of the CS group was significantly higher than that of the other treatment groups (*p <* 0.01). The results showed that *B. velezensis* CB13 treatment significantly increased the diversity of soil bacteria. The fungal diversity of the soil after *B. velezensis* CB13 treatment was not significantly different from that of the control (*p* > 0.05), whereas the fungal diversity of soil inoculated with *S. rolfsii* decreased significantly compared to that of the control (*p <* 0.05).

At the phylum level, the composition and abundance of soil bacterial and fungal flora in the peanut root system were analyzed (Figures 6E,F; Supplementary Tables S5, S6). The predominant bacterial phyla (>1%) were Actinobacteriota, Proteobacteria, Chloroflexi, Acidobacteriota, Bacteroidota, Firmicutes, Patescibacteria, Gemmatimonadota, and Myxococcota. Inoculation with *B. velezensis* CB13 significantly decreased the abundance of Actinobacteriota and significantly increased the abundance of Proteobacteria, Acidobacteriota, Firmicutes, Gemmatimonadota, and Myxococcota. The predominant fungal phyla (>1%) were Ascomycota, Basidiomycota, unclassified_k_Fungi, Mortierellomycota, and Olpidiomycota. Inoculation with *B. velezensis* CB13 significantly decreased the abundance of Olpidiomycota (*p* < 0.05). Differential abundance analysis by using DESeq2, we confirmed that *B. velezensis* CB13 significantly increased the abundance of Proteobacteria, Firmicutes, Gemmatimonadota, and Myxococcota and significantly decreased the abundance of Actinobacteriota and Olpidiomycota (*p* < 0.05; Supplementary Table S7).

**Figure 7:**
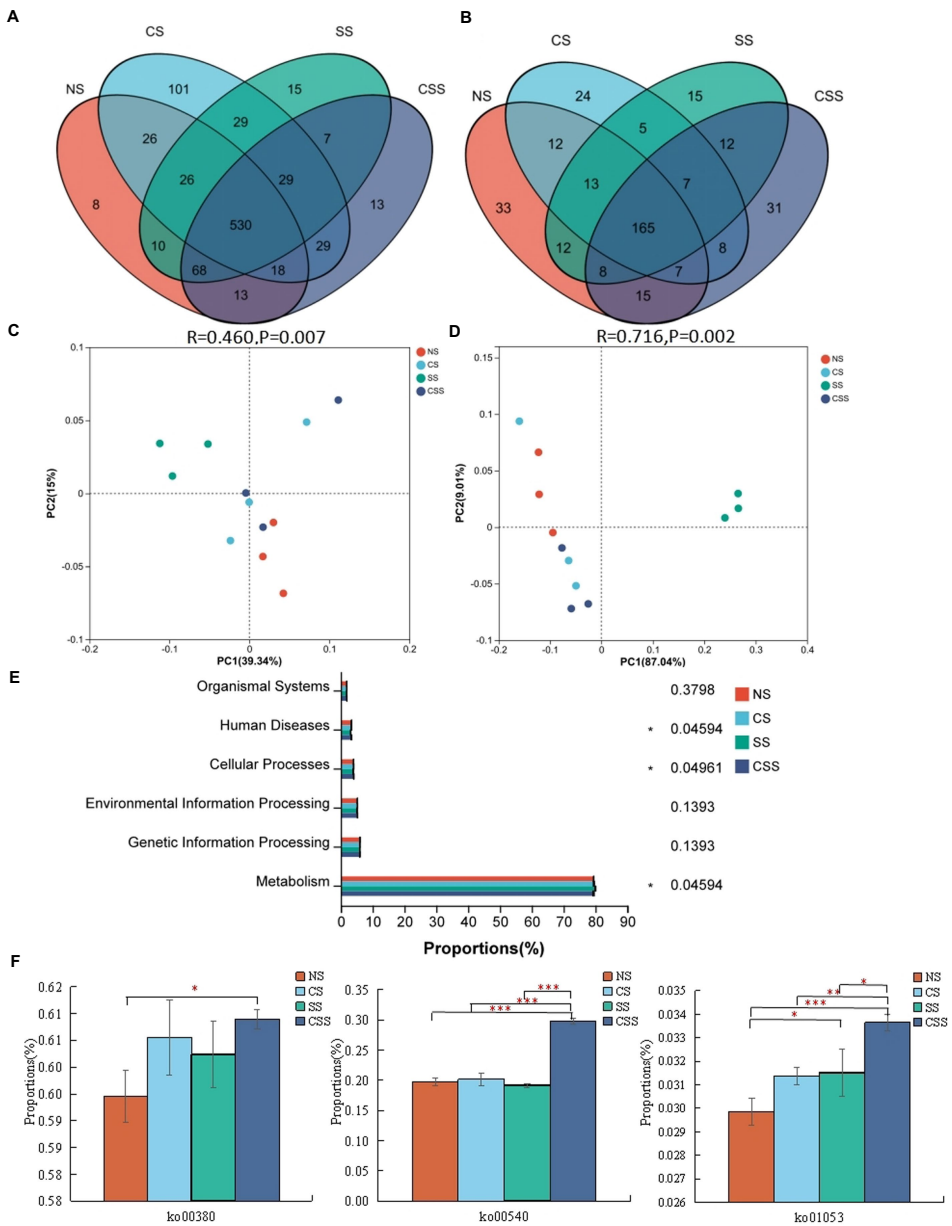
Comparative analysis and Functional analysis of peanut rhizosphere soil microbial community. **(A)** Venn analysis of soil bacterial composition at the genus level. **(B)** Venn analysis of soil fungal composition at the genus level; **(C)** Principal coordinate analysis (PCoA) plot showing bacteria community structure variation between the samples. **(D)** PCoA plot showing variation in fungal community structure among the samples; each symbol represents a sample, and the closer the two sample points, the more similar the species composition of the two samples; **(E)** The functional composition and relative abundance of bacteria in the primary functional layer based on the Kyoto Encyclopedia of Genes and Genomes (KEGG) pathway. The right axis is the *p-*value (*p < 0.05); **(F)** Function analysis of bacteria in the third functional layer based on the KEGG pathway; ko00380: tryptophan metabolism; ko00540: lipopolysaccharide biosynthesis; ko01053: biosynthesis of siderophore group non-ribosomal peptides; Differences between the means in each group were compared using a Wilcoxon rank-sum test (*p < 0.05, **p < 0.01, and ***p < 0.001). NS, uninoculated soil; CS, soil inoculated with *Bacillus velezensis* CB13; SS, soil inoculated with *Sclerotium rolfsii*; CSS, soil inoculated with *B. velezensis* CB13 and *S. rolfsii*.

Venn analysis revealed that the soil samples from different groups had 530 bacterial genera and 165 fungal genera in common (Figures 7A,B). The 10 dominant genera identified in the soil samples were selected for relative proportion variance (Supplementary Figure S2). Compared to that in the uninoculated soil, the proportion of the 10 dominant bacterial genera did not change significantly after *B. velezensis* CB13 treatment (*p* > 0.05). Regarding fungi, after *B. velezensis* CB13 treatment, the proportion of *Pseudogymnoascus* increased compared with that in the uninoculated soil (*p <* 0.05). Differential abundance analysis showed that there were no significant differences in the proportion of the 10 dominant bacterial and fungal genera between the uninoculated and inoculation with *B. velezensis* CB13 soil (*p* > 0.05). PCoA confirmed that the samples from soil inoculated with *S. rolfsii* clustered together, indicating that *S. rolfsii* influences rhizosphere microbiome community structure (Figures 7C,D). Contrastingly, the results for other samples that were separately clustered suggested that *B. velezensis* CB13 could alter the structure and maintain the stability of rhizosphere microbiome community structure.

The functional composition and proportion of the microbial diversity in the peanut rhizosphere soil were analyzed in different samples. According to the Kyoto Encyclopedia of Genes and Genomes (KEGG) pathway, the primary functional layer consists of cellular processes, environmental and genetic information processing, human diseases, metabolism, and organism system (Figure 7E). The different sample groups significantly differed in terms of human diseases, cellular processes, and metabolism. Further functional analysis showed that the secondary functional layer of genes predominantly comprised 46 subfunctions, including global and overview maps, carbohydrate 337 metabolism, amino acid metabolism, and energy metabolism, etc. (Supplementary Table S8). Additionally, we focused on the pathways related to disease resistance in the third functional layer (Figure 7F). Compared with that in the uninoculated soil, the functional proportion of genes involved in lipopolysaccharide biosynthesis, biosynthesis of siderophore group non-ribosomal peptides, and tryptophan metabolism in the soil inoculated with *B. velezensis* CB13 and *S. rolfsii* increased significantly (*p <* 0.05).

Based on the MetaCyc pathway, the gene functions of fungi comprised 74 pathways, including aerobic respiration, the glyoxylate cycle, the pentose phosphate pathway (non-oxidative branch), and guanosine nucleotide degradation (Supplementary Table S9). After *B. velezensis* CB13 treatment, the proportion of the functional pathways of NAD/NADH phosphorylation and dephosphorylation and 4-amino-2-methyl-5-phosphomethylpyrimidine biosynthesis (yeast) increased significantly (*p <* 0.05), and the proportion of functional pathways of GDP-mannose biosynthesis, adenine and adenosine salvage III, phospholipid remodeling (phosphatidylethanolamine, yeast), and phospholipases decreased significantly (*p <* 0.05). Compared with that in the uninoculated soil, the proportion of 11 pathways in the soil inoculated with *S. rolfsii* was significantly higher (*p* < 0.05), including glyoxylate cycle, fatty acid, and beta-oxidation (peroxisome, yeast), etc. In the soil inoculated with *S. rolfsii*, the proportion of 16 pathways, including pentose phosphate pathway (non-oxidative branch), D-myo-inositol (1,4,5)-trisphosphate biosynthesis, etc. were significantly lower compared to the uninoculated soil (*p* < 0.05).

### 3.7. Quantitative analysis of *bacillus* spp. and *Sclerotium rolfsii* in peanut rhizosphere soil

In order to determine the content of *Bacillus* spp. and *S. rolfsii*, we designed the specific primers and confirmed the specificity by electrophoresis analysis. The results indicated that there was a single and clear band amplified by B1F/B1R in four treatment groups, and there was no visible band observed amplified by SRITSF/SRITSR in uninoculated *S. rolfsii* groups (Supplementary Figure S3). Thus, these primers could be accepted and used for the quantification of *Bacillus* spp. and *S. rolfsii* in peanut rhizosphere soil. The qPCR results of *Bacillus* spp. and *S. rolfsii* (Figures 4E,F) show that the population of *Bacillus* spp. in the CS and CSS groups were significantly higher than those in the NS and SS groups, respectively (*p* < 0.05), indicating that *B. velezensis* CB13 stably colonized or increased the content of *Bacillus* spp. in the soil. The population of *S. rolfsii* in SS group was significantly higher than that in the CSS group (*p* < 0.05), indicating that the biocontrol bacterium *B. velezensis* CB13 significantly inhibited the reproduction of *S. rolfsii* in the soil.

## 4. Discussion

In this study, we comprehensively evaluated the biocontrol potential of *B. velezensis* CB13 based on bacteriostatic activity, colonization ability, induced defense enzyme activity, and its effect on soil microorganisms. *B. velezensis* CB13 was isolated from pig biogas slurry and was effective in reducing *S. rolfsii* radial growth. In pot trials, *B. velezensis* CB13 significantly controlled peanut stem rot; this effect in the pot trials was significantly greater than that previously reported for *B. velezensis* LHSB1 (Chen et al., 2020a,b), *B. velezensis* DPT-03 (Peng et al., 2022), *Bacillus sinensis* ZHX-10 (Zhang et al., 2020), *Bacillus amyloliticus* LX-J1 (Yang et al., 2017), and *Bacillus amyloliquefaciens* 41B-1 (Li, 2018). Our results demonstrate the utility of *B. velezensis* CB13 as a biocontrol agent against peanut stem rot.

Plant and root colonization and as well as the formation of dominant populations are preconditions for effective disease control by biocontrol agents (Christopher et al., 2021). Haggag and Timmusk, (2008) compared two strains of bacteria isolated from peanut root soil that inhibited the growth of *Aspergillus niger*, *Paenibacillus polymyxa* B5, and *Paenibacillus polymyxa* B6. Although the two strains produced antibiotics at the same rate, the more abundantly colonizing B5 was more effective in controlling peanut crown rot. Lu et al. (2015) found that in pot experiments, the colonizing ability of *Trichoderma virensis* TV41 in the rhizosphere and root surface of watermelon was significantly better than that of *Fusarium oxysporum* FWO and helped in preventing and controlling the occurrence of watermelon *Fusarium wilt*. These observations demonstrate the importance of effective colonization for biological control. This was confirmed in the current study through chloramphenicol screening of GFP-tagged strain CB13 in peanut roots and rhizosphere soil. CB13-GFP stably colonized the peanut roots and soil, and 10^4^ CFU/g and 10^8^ CFU/g of CB13-GFP strain could still be detected in peanut roots and soil, respectively, after 50 days. The qPCR results deciphered that *B. velezensis* CB13 stably colonized the soil or increased the population of *Bacillus* spp. in the soil and *B. velezensis* CB13 significantly inhibited the reproduction of *S. rolfsii* in soil.

The resistance posed by the entire plant when infected by pathogens is called systemic acquired resistance. This resistance can improve the plant’s defense against a variety of pathogens, nematodes, and even parasitic plants. Furthermore, beneficial microorganisms can induce systemic resistance in plants. Induced systemic resistance does not necessarily directly activate the defense response, but the plant shows faster and/or stronger cellular defense response activation upon invasion by pathogens (Figueredo et al., 2014). Induced systemic resistance involves a series of defense responses that spread from the induction site to the distal parts of the plant. The responses include plant hormone-mediated signal transduction, production of plant defensins, oxidative stress protection, and production of plant defense-related enzymes including PAL, PPO, CAT, POD, SOD, CHI, and GLU. *Bacillus* induces disease resistance in plants by increasing the activities of defense enzymes (Mielich-Suss and Lopez, 2015; Wang et al., 2017). For example, the host enzymes induced by *B. subtilis* include POD, PPO, SOD, and others, whose increased synthesis induces systemic resistance against early and late blight in tomato seedlings (Chowdappa et al., 2013). *B. amylolyticus* HRH317 increases the activities of SOD, CAT, PAL, POD, PPO, CHI, and GLU in maize leaves, which enhances resistance (Qin, 2020). *B. velezensis* FJ17-4 induces SOD, POD, CAT, PAL, and PPO defense enzyme activities in cucumber roots and enhances the systemic disease resistance of cucumber (Lan, 2021). In this study, the activities of PAL, PPO, POD, CAT, APX, and CHI in peanut leaves inoculated with both *B. velezensis* CB13 and *S. rolfsii* were significantly higher than those in peanut leaves inoculated only with *S. rolfsii*. Peanut plants treated with *B. velezensis* CB13 showed a stronger defense response against *S. rolfsii* infection. In addition, in peanuts treated with *B. velezensis* CB13, the activities of SOD and GLU after 12 h of *S. rolfsii* inoculation were significantly higher than those in peanut plants inoculated only with *S. rolfsii*, indicating that peanut plants treated with *B. velezensis* CB13 had faster defense responses to *S. rolfsii* infection.

Microbial diversity is not only an important feature of healthy soil but also a direct reflection of the disease resistance of soil. The higher the soil microbial diversity, the higher the species richness and evenness, the more stable the microbial ecosystem, and the stronger the ability to resist pathogenic microorganisms (Garbeva et al., 2004). Contrastingly, the simplification of soil microbial diversity could contribute to soil sickness and increased plant pests and diseases (Mustafa et al., 2019; Chen et al., 2020b). In this study, *B. velezensis* CB13 significantly increased the species diversity of bacteria in soil, which is consistent with the findings of other studies. Cucumber roots treated with *B. velezensis* FJ17-4 shows increased soil microbial community diversity (Lan, 2021). *Bacillus amylolyticus* controls tobacco bacterial wilt by changing the soil microbial structure and abundance of plant roots (Bo et al., 2016). At the phylum level, inoculation with *B. velezensis* CB13 significantly increased the abundance of Proteobacteria, Acidobacteriota, Firmicutes, Gemmatimonadota, and Myxococcota. Firmicutes and Proteobacteria are known to be fast-growing organisms that prefer nutrient-rich environments and are involved in the degradation of complex organic compounds (Li et al., 2020). Acidobacteriota are important microorganisms in soil ecosystems that maintain the ecological balance of soil and plant growth promotion (Kalam et al., 2020). Several lineages of Gemmatimonadota are capable of anoxygenic photosynthesis (Izabela et al., 2022), and their abundance is positively correlated with total carbon, nitrogen, and phosphorus contents in soil (Deng et al., 2020). The functional analysis of microbial community showed that combined inoculation with *B. velezensis* CB13 and *S. rolfsii* significantly enhanced lipopolysaccharide biosynthesis, the biosynthesis of siderophore group non-ribosomal peptides, and the tryptophan metabolism pathways of peanut rhizosphere microbial community. The enhancement of these pathways contributed to the increase in defense enzyme activity in peanut plants and resistance to pathogenic. Some microorganisms can synthesize indole-3-acetic acid (IAA) from tryptophan. IAA, lipopolysaccharide, and siderophore have been demonstrated to be elicitors that induce plant systemic resistance (Loon et al., 1998). These reports suggest that treatment with *B. velezensis* CB13 not only improves the soil microbial community diversity of peanut roots but also increases the relative abundance of beneficial bacteria, promotes soil fertility, improves soil disease resistance, and changes the composition of the soil microbial community and soil environment in a beneficial manner.

In conclusion, *B. velezensis* CB13 showed a significant inhibitory effect on the radial growth of *S. rolfsii*, a pathogen that causes peanut stem rot, and a significant biocontrol effect in pot experiments. In addition, *B. velezensis* CB13 controlled stem rot through different or composite mechanisms, including the inhibition of plant pathogen growth, induction of systemic resistance in plants, soil colonization, and improvements in the microbial diversity and fertility of the peanut rhizosphere. Accordingly, antifungal substances of *B. velezensis* CB13 and strategies for their application need to be further explored.

## Data availability statement

The datasets presented in this study can be found in online repositories. The names of the repository/repositories and accession number(s) can be found below: PRJNA900388, OP430814, OP889276, OP889277, and OP889278.

## Author contributions

MJ and XL: conceptualization. SJ and JC: data curation. SJ and XL: formal analysis. SJ and XY: sampling. SJ and HD: writing–original draft preparation. MJ, XL, and JC: writing–review and editing. CS and XL: supervision. CS and JC: project administration. All authors have read and agreed to the published version of the manuscript.

## Funding

This work was supported by the National Modern Agriculture Industry Technology System Construction Project (CARS-18), Nature Science Foundation of Liaoning (2018010565–301), and National Natural Science Foundation of China (32172454).

## Conflict of interest

The authors declare that the research was conducted in the absence of any commercial or financial relationships that could be construed as a potential conflict of interest.

## Publisher’s note

All claims expressed in this article are solely those of the authors and do not necessarily represent those of their affiliated organizations, or those of the publisher, the editors and the reviewers. Any product that may be evaluated in this article, or claim that may be made by its manufacturer, is not guaranteed or endorsed by the publisher.

## Footnotes

^1^ http://www.ncbi.nlm.nih.gov/

^2^ http://rdp.cme.msu.edu/

^3^ http://www.arb-silva.de

^4^ https://unite.ut.ee/

^5^ https://github.com/picrust/picrust2/

## References

[ref1] Bo W. Xiao W. Liang Y. Huan Y. Heng Z. Qiu Y. M.. (2016). Effects of *bacillus amyloliquefaciens* ZM9 on bacterial wilt and rhizosphere microbial communities of tobacoo. Appl. Soil Ecol. 103, 1–12. doi: 10.1016/j.apsoil.2016.03.002

[ref2] Bouchenak M. Lamri-Senhadji M. (2013). Nutritional quality of legumes and their role in cardiometabolic risk prevention: a review. J. Med. Food 16, 185–198. doi: 10.1089/jmf.2011.0238, PMID: 23398387

[ref3] Cao F. M. Yang X. H. Ma M. C. Chen H. J. Shen D. L. Li J. (2014). Advances in the identification of *Bacillus subtilis* and closely related species. Microbiol. China 8, 8–18. doi: 10.1007/s12602-016-9208-z, PMID: 26898909

[ref4] Chen K. R. Ren L. Xu L. Chen W. Liu F. Fang X. P. (2018). Research progress on peanut southern stem rot caused by *Sclerotium rolfsii*. Chin. J. Oil. Crop. Sci 40, 302–308. doi: 10.7505/j.issn.1007-9084.2018.02.018

[ref5] Chen L. Wu Y. D. Chong X. Y. Xin Q. H. Wang D. X. Bian K. (2020a). Seed-borne endophytic *bacillus velezensis* LHSB1 mediate the biocontrol of peanut stem rot caused by *Sclerotium rolfsii*. J. Appl. Microbiol. 128, 803–813. doi: 10.1111/jam.14508, PMID: 31705716

[ref6] Chen M. N. Zhang J. C. Liu H. Wang M. Pan L. J. Chen N. (2020b). Long-term continuously monocropped peanut significantly disturbed the balance of soil fungal communities. J. Microbiol. 58, 563–573. doi: 10.1007/s12275-020-9573-x, PMID: 32329018

[ref7] Chen S. F. Zhou Y. Q. Chen Y. R. Gu J. (2018). fastp: an ultra-fast all-in-one FASTQ preprocessor. Bioinformatics 34, i884–i890. doi: 10.1093/bioinformatics/bty560, PMID: 30423086 PMC6129281

[ref8] Chowdappa P. Kumar S. M. Lakshmi M. J. Upreti K. K. (2013). Growth stimulation and induction of systemic resistance in tomato against early and late blight by *Bacillus subtilis* OTPB1 or *Trichoderma harzianum* OTPB3. Biol. Control 65, 109–117. doi: 10.1016/j.biocontrol.2012.11.009

[ref9] Christopher B. Mathilde N. C. Ákos T. K. (2021). Molecular aspects of plant growth promotion and protection by *Bacillus subtilis*. Mol. Plant-Microbe Interact. 34, 15–25. doi: 10.1094/MPMI-08-20-0225-CR, PMID: 32986513

[ref10] Clarke K. R Warwick R. M. (2001). Change in Marine Communities: An Approach to Statistical Analysis and Interpretation, 2nd Edn PRIMER-E, Plymouth.

[ref11] Cole J. R. Wang Q. Cardenas E. Fish J. Chai B. Farris R. J.. (2009). The ribosomal database project: improved alignments and new tools for rRNA analysis. Nucl. Acids. Res 37, D141–D145. doi: 10.1093/nar/gkn879, PMID: 19004872 PMC2686447

[ref12] Courchesne F. Gobran G. R. (1997). Mineralogical variations of bulk and rhizosphere soils from a Norway spruce stand. Soil Sci. Soc. Am. J. 61, 1245–1249. doi: 10.2136/sssaj1997.03615995006100040034x

[ref13] Deng J. J. Bai X. J. Zhou Y. B. Zhu W. X. Yin Y. (2020). Variations of soil microbial communities accompanied by different vegetation restoration in an open-cut iron mining area. Sci. Total Environ. 704:135243. doi: 10.1016/j.scitotenv.2019.135243, PMID: 31787305

[ref14] Djordje F. Ivica D. Tanja B. Jelena L. Slaviša S. (2018). Biological control of plant pathogens by *bacillus* species. J. Biotechnol. 285, 44–55. doi: 10.1016/j.jbiotec.2018.07.044 30172784

[ref15] Edgar R. C. (2013). UPARSE: highly accurate OTU sequences from microbial amplicon reads. Nat. Methods 10, 996–998. doi: 10.1038/nmeth.2604, PMID: 23955772

[ref16] Fan P. M. (2021). *Germplasm assessment and transcriptomics and metabolomics analysis for resistance*. Master dissertation, Beijing: Chinese Academy of Agricultural Sciences.

[ref17] Felsenstein J. (1985). Confidence limits on phylogenies an approach using the bootstrap. Evolution 39, 783–791. doi: 10.1111/j.1558-5646, PMID: 28561359

[ref18] Figueredo M. S. Tonelli M. L. Taurian T. Angelini J. Ibanez F. Valetti L.. (2014). Interrelationships between *bacillus* sp. CHEP5 and Bradyrhizobium sp. SEMIA6144 in the induced systemic resistance against *Sclerotium rolfsii* and symbiosis on peanut plants. J. Biosci. 39, 877–885. doi: 10.1007/s12038-014-9470-8, PMID: 25431416

[ref19] Garbeva P. Veen J. A. Elsas J. D. (2004). Microbial diversity in soil: selection of microbial populations by plant and soil type and implications for disease suppressiveness. Annu. Rev. Phytopathol. 42, 243–270. doi: 10.1146/annurev.phyto.42.012604.135455, PMID: 15283667

[ref20] Haggag W. M. Timmusk S. (2008). Colonization of peanut roots by biofifilm-forming *Paenibacillus polymyxa* initiates biocontrol against crown rot disease. J. Appl. Microbiol. 104, 961–969. doi: 10.1111/j.1365-2672.2007.03611.x, PMID: 18005030

[ref21] Höfer V. Dölle-Bierke S. Reiner R. Worm M. (2022). Influence of nut and peanut allergy on the quality of life of adults. Hautarzt 73, 186–194. doi: 10.1007/s00105-021-04939-6, PMID: 35072740

[ref22] Izabela M. Kasia P. Michal K. (2022). *Phylum Gemmatimonadota* and its role in the environment. Microorganisms 10:151. doi: 10.3390/microorganisms10010151, PMID: 35056600 PMC8779627

[ref23] Jacob S. Sajjalaguddam R. R. Sudini H. K. (2018). Streptomyces sp. RP1A-12 mediated control of peanut stem rot caused by *Sclerotium rolfsii*. J. Integr. Agr 17, 892–900. doi: 10.1016/S2095-3119(17)61816-1

[ref24] Jia S. He Y. Z. Yu T. H. Zhao C. Zuo L. L. Song C. (2020). Ultrastructure observation in the fat body of *Antheraea Pernyi* larvae infected by the parasitic nematode. Nor. Seri 41, 33–36. doi: 10.3969/j.issn.1673-9922.2020.03.007

[ref25] Jing C. L. Xu Z. C. Zou P. Tang Q. Li Y. Q. You X. W.. (2019). Coastal halophytes alter properties and microbial community structure of the saline soils in the Yellow River Delta. China. Appl. Soil Ecol. 134, 1–7. doi: 10.1016/j.apsoil.2018.10.009

[ref26] Jones J. B. Provost M. Keaver L. Breen C. Ludy M. J. Mattes R. D. (2014). A randomized trial on the effects offlavorings on the health benefits of daily peanut consumption. Am. J. Clin. Nutr. 99, 490–496. doi: 10.3945/ajcn.113.069401, PMID: 24351876

[ref27] Kalam S. Basu A. Ahmad I. Sayyed R. Z. El-Enshasy H. A. Dailin D. J.. (2020). Recent understanding of soil Acidobacteria and their ecological significance: a critical review. Front. Microbiol. 11, 1–15. doi: 10.3389/fmicb.2020.580024, PMID: 33193209 PMC7661733

[ref28] Ki J. S. Zhang W. Qian P. Y. (2009). Discovery of marine *bacillus* species by 16S rRNA and rpo B comparisons and their usefulness for species identification. J. Microbiol. Methods 77, 48–57. doi: 10.1016/j.mimet.2009.01.003, PMID: 19166882

[ref29] Kim J. Rohlf F. J. Sokal R. R. (1993). The accuracy of phylogenetic estimation using the neighbor-joining method. Evolution 47, 471–486. doi: 10.1111/j.1558-5646.1993.tb02107.x 28568714

[ref30] Lan C. Z. (2021). *LAMP detection of Fusarium oxysporum f. sp. cucumerinum causing cucumber Fusarium wilt and biological control mechanism of the Bacillus velezensis FJ17-4*. Master dissertation, Nanchang: Jiangxi Agricultural University.

[ref31] Le C. N. Hoang T. K. Thai T. H. Tran T. L. Phan T. P. N. Raaijmakers J. M. (2018). Isolation, characterization and comparative analysis of plant-associated bacteria for suppression of soil-borne diseases of field-grown groundnut in Vietnam. Biol. Control 121, 256–262. doi: 10.1016/j.biocontrol.2018.03.014

[ref32] Li R. (2011). Competent preparation and plasmid transformation of *Bacillus subtilis*. Biotechnol. Bull. 27, 105–114. doi: 10.1007/s10255-011-0044-3

[ref33] Li C. (2018). *Control effect and mechanism of Bacillus amyloliquefaciens 41B-1 on Sclerotium rolfsii*. Master dissertation, Nanjing: Agricultural University.

[ref34] Li M. R. Sen G. Chi-Tang H. Naisheng B. (2022). Review on chemical compositions and biological activities of peanut (*Arachis hypogeae* L.). J. Food Biochem. 46:e14119. doi: 10.1111/jfbc.14119, PMID: 35253912

[ref35] Li L. Y. Song W. D. Zhang F. Lei Y. Wan L. Y. Huan D. Y.. (2017). Artificial inoculation technique for peanut stem rot caused by *Sclerotium rolfsii* and evaluation resistance of peanut seeding in greenhouse. Oil. Crop. Sci. 39, 687–692. doi: 10.7505/j.issn.1007-9084.2017.05.014

[ref36] Li W. X. Zhang Y. P. Mao W. Wang C. S. Yin S. X. (2020). Functional potential differences between Firmicutes and Proteobacteria in response to manure amendment in a reclaimed soil. Can. J. Microbiol. 66, 689–697. doi: 10.1139/cjm-2020-0143, PMID: 32717168

[ref37] Liu F. Y. Yang S. Xu F. H. Zhang Z. Lu Y. F. Zhang J. M.. (2022). Characteristics of biological control and mechanisms of *Pseudomonas chlororaphis* zm-1 against peanut stem rot. BMC Microbiol. 22, 9–13. doi: 10.1186/s12866-021-02420-x, PMID: 34986788 PMC8729073

[ref38] Loon L. C. V. Pakker P. A. Pieterse C. M. (1998). Systemic resistance induced by rhizosphere bacteria. Annu. Rev. Phytopathol. 36, 453–483. doi: 10.1146/annurev.phyto.36.1.453 15012509

[ref39] Love M. I. Huber W. Anders S. (2014). Moderated estimation of fold change and dispersion for RNA-seq data with DESeq2. Genome Biol. 15:550. doi: 10.1186/s13059-014-0550-8, PMID: 25516281 PMC4302049

[ref40] Lu P. Jiang K. Hao Y. Q. Chu W. Y. Xu Y. D. Yang J. Y.. (2021). Profiles of *bacillus* spp. isolated from the rhizosphere of *Suaeda glauca* and their potential to romote plant growth and suppress *Yungal phytopathogens*. J. Microbiol. Biotechnol. 31, 1231–1240. doi: 10.4014/jmb.2105.05010, PMID: 34261851 PMC9706026

[ref41] Lu C. Y. Ma Y. Wang Q. Wang Q. J. Wang G. F. Mei Y.. (2015). Effects of *Trichoderma viride* TV41 on spatial distribution of *Fusarium oxysporum* FW0 around watermelon plant and fusarium wilt contro. Microbiol. China 42, 2159–2167. doi: 10.13344/j.microbiol.china.150405

[ref42] Luo Z. L. Cui R. J. Chavarro C. Tseng Y. C. Zhou H. Peng Z.. (2020). Mapping quantitative trait loci(QTLs) and estimating the epistasis controlling stem rot resistance in cultivated peanut (*Arachis hypogaea*). Theor. Appl. Genet. 133, 1201–1212. doi: 10.1007/s00122-020-03542-y, PMID: 31974667

[ref43] Mielich-Suss B. Lopez D. (2015). Molecular mechanisms involved in *Bacillus subtilis* biofilm formation. Environ. Microbiol. 17, 555–565. doi: 10.1111/1462-2920.12527, PMID: 24909922 PMC4188541

[ref44] Motlagh M. R. S. Farokhzad M. Kaviani B. Kulus D. (2022). Endophytic fungi as potential biocontrol agents against *Sclerotium rolfsii* Sacc.-the causal agent of Peanut white stem rot disease. Cells 11:2643. doi: 10.3390/cells11172643, PMID: 36078051 PMC9454559

[ref45] Mustafa M. Mayes S. Massawe F. (2019). Crop diversification through a wider use of underutilised crops: a strategy to ensure food and nutrition security in the face of climate change. Sustain. Solut. Food Security 7, 125–149. doi: 10.1007/978-3-319-77878-5_7

[ref46] Pan M. S. Guo W. Y. Zhou L. Z. Deng L. Miao J. L. Xu H. G.. (2022). Evaluation of efficacy in field and mechanism of *Bacillus velezensis* agents for controling peanut stem rot caused buy *Sclerotium rolfsii*. J. Agric. Sci. Technol. 24, 130–136. doi: 10.13304/j.nykjdb.2022.0122

[ref47] Peng Q. C. Huang D. L. Zhang Z. P. Wei H. Wu S. F. Wu Y.. (2022). The biocontrol effect of *Bacillus velezensis* DPT-03 on *Sclerotium rolfsii* in peanut. J. Henan Agri. Sci. 51, 97–103. doi: 10.15933/j.cnki.1004-3268.2022.02.011

[ref48] Pravi V. Jeeva M. L. Archana P. V. (2014). Rapid and sensitive detection of *Sclerotium rolfsii* associated with collar rot disease of *Amorphophallus peaoniifolius* by species-spdcific polymerase chain reaction assay. Mol. Biotechnol. 56, 787–794. doi: 10.1007/s12033-014-9757-x, PMID: 24788585

[ref49] Qin N. (2020). *The mechanism of inhibiting Fusarium moniliforme to infect maize seedlings with Bacillus amyloliquefaciens HRH317*. Master dissertation, Jinzhong: Shanxi Agricultural University.

[ref50] Saxena A. K. Kumar M. Chakdar H. Anuroopa N. Bagyaraj D. J. (2020). *Bacillus* species in soil as a natural resource for plant health and nutrition. J. Appl. Microbiol. 128, 1583–1594. doi: 10.1111/jam.14506, PMID: 31705597

[ref51] Scheeomaker J. W. A. Kassteele J. V. D. (2014). Effects of chemical control agents and microbial biocontrol agents on numbers of non-target microbial soil organisms: a meta-analysis. Biocontrol Sci. Tech. 21, 1225–1242. doi: 10.1080/09583157.2011.594952

[ref52] Schloss P. D. Westcott S. L. Ryabin T. Hall I. R. Hartmann M. Hollister E. B.. (2009). Introducing mothur: open-source, platform-independent, community-supported software for describing and comparing microbial communities. Appl. Environ. Microb 75, 7537–7541. doi: 10.1128/AEM.01541-09, PMID: 19801464 PMC2786419

[ref53] Sconyers L. E. Brenneman T. B. Stevenson K. L. Mullinix B. G. (2007). Effects of row pattern, seeding rate, and inoculation date on fungicide efficacy and development of Peanut stem rot. Plant Dis. 91, 273–278. doi: 10.1094/PDIS-91-3-0273, PMID: 30780560

[ref1001] Shan H. Zhao M. Chen D. Cheng J. Li J. Feng Z.. (2013). Biocontrol of rice blast by the phenaminomethylacetic acid producer of Bacillus methylotrophicus strain BC79. Crop. Prot. 44, 29–37. doi: 10.1016/j.cropro.2012.10.012, PMID: 19801464

[ref54] Standish J. R. Culbreath A. K. Branch W. D. Brenneman T. B. (2019). Disease and yield response of a stem-rot-resistant and susceptible Peanut cultivar under varying fungicide inputs. Plant Dis. 103, 2781–2785. doi: 10.1094/PDIS-04-19-0771-RE, PMID: 31469362

[ref55] Tanja M. Steven L. S. (2011). FLASH: fast length adjustment of short reads to improve genome assemblies. Bioinformatics 27, 2957–2963. doi: 10.1093/bioinformatics/btr507, PMID: 21903629 PMC3198573

[ref56] Thompson J. D. Gibson T. J. Plewniak F. Jeanmougin F. Higgins D. G. (1997). The CLUSTAL_X windows interface: flflexible strategies for multiple sequence alignment aided by quality analysis tools. Nucleic Acids Res. 25, 4876–4882. doi: 10.1093/nar/25.24.4876, PMID: 9396791 PMC147148

[ref57] Timper P. Minton N. A. Johnson A. W. Brenneman T. B. Culbreath A. K. Burton G. W.. (2001). Influence of cropping systems on stem rot (*Sclerotium rolfsii*), *Meloidogyne arenaria*, and the nematode antagonist *Pasteuria penetrans* in Peanut. Plant Dis. 85, 767–772. doi: 10.1094/PDIS.2001.85.7.767, PMID: 30823204

[ref58] Toomer O. T. (2018). Nutritional chemistry of the peanut (*Arachis hypogaea*). Crit. Rev. Food. Sci 58, 3042–3053. doi: 10.1080/10408398.2017.1339015 28662347

[ref59] Wang W. M. Han L. Z. Wang H. (2020). Effects of two *bacillus* spp. strains on the frowth of peanut seeding and microbial community structure in rhizosphere soil. Microbiol. China 47, 3551–3563. doi: 10.13344/j.microbiol.china.190977

[ref60] Wang H. Wang Y. X. Yang R. J. (2017). Recent progress in *Bacillus subtilis* spore-surface display: concept, progress, and future. Appl. Microbiol. Biotechnol. 101, 933–949. doi: 10.1007/s00253-016-8080-9, PMID: 28062973

[ref61] Xu N. Tan G. G. Wang H. Y. Gai X. P. (2016). Effect of biochar additions to soil on nitrogen leaching, microbial biomass and bacterial community structure. Eur. J. Soil Biol. 74, 1–8. doi: 10.13227/j.hjkx.202003113

[ref62] Yan L. Y. Wang Z. H. Song W. D. Fan P. M. Kang Y. P. Lei Y.. (2021). Genome sequencing and comparative genomic analysis of highly and weakly aggressive strains of *Sclerotium rolfsii*, the causal agent of peanut stem rot. BMC Genomics 22:276. doi: 10.1186/s12864-021-07534-0, PMID: 33863285 PMC8052761

[ref63] Yang N. Li L. Y. Sun B. B. Xiao Y. Xu Y. P. (2017). Study on the effect of *Bacillus amyloliquefaciens* LX-J1 and its fertilizer on the control of peanut white silk disease. J. Gen. Sci. Tec 25, 73–76. doi: 10.16663/j.cnki.lskj.20170809.001

[ref64] Yu D. Yan L. Y. Song W. D. Kang Y. P. Lei Y. Chen Y. N.. (2022). Progress on pathogenicity differentiation in *Sclerotiun roolfsii* isolates from peanut. Chin. J. Oil Crop Sci. 44, 930–936. doi: 10.19802/j.issn.1007-9084.2021256

[ref65] Zhang X. Xu M. L. Guo Z. Q. Yu J. Wu J. X. Yu J. L.. (2020). Isolation and identification of *Bacillus sinensis* ZHX-10 and analysison its biological control activities against *Sclerotium rolfsii*. Chin. J. Oil Crop Sci. 42, 674–680. doi: 10.19802/j.issn.1007-9084.2019207

[ref66] Zhou F. Zhou Y. D. Zhang Y. T. Li S. W. Gao M. R. Ma B. M.. (2022). Biological characteristics of peanut sclerotium blight and its sensitivity to fungicides. China Plant Protection 42, 19–23. doi: 10.15904/j.cnki.hnny.2022.29.005

[ref67] Zhu X. F. (2011). *Experimental techniques of modern microbiology*. Master dissertation, Hangzhou, Zhejiang University Press.

