# Supplementary material for: Evaluation of efficacy and mechanism of Bacillus velezensis CB13 for controlling peanut stem rot caused by Sclerotium rolfsii
Source: Front Microbiol. 2023 Feb 16;14:1111965. doi: 10.3389/fmicb.2023.1111965 (PMC9978184; doi:10.3389/fmicb.2023.1111965)
Supplement: Supplementary file 1 [file Data_Sheet_1.docx]

Supplementary Material

Evaluation of efficacy and mechanism of *Bacillus velezensis* CB13 for controling peanut stem rot caused by *Sclerotium rolfsii*

Shu Jia^1,2^, Ce Song^2^, Hai Dong^3^, Xujie Yang^1^, Xinghai Li^1*^, Mingshan Ji^1*^, Jin Chu^3*^

*** Correspondence:**

Xinghai Li:E-mail: [xinghai30@163.com](mailto:xinghai30@163.com) or

Mingshan Ji:E-mail: jimingshan@163.com or

Jin Chu: E-mail: chujin86@vip.qq.com

# Supplementary Figures and Tables

## Supplementary Figures

A

B


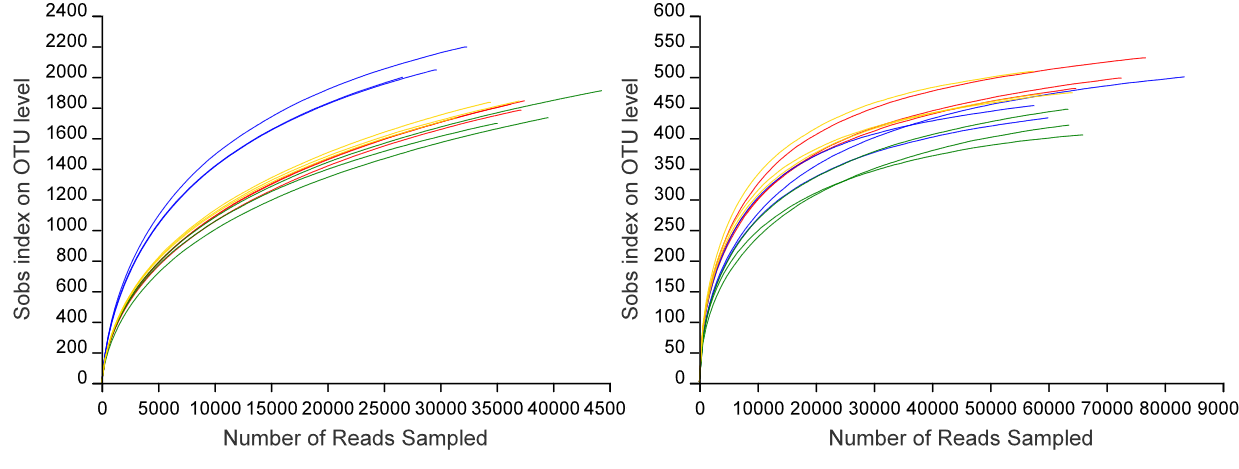


**Supplementary Figure 1.** Rarefaction curves of the bacteria (A) and fungi(B) according to OTU.

# 1.2

A

B

# Supplementary Figure 2. Comparative analysis of peanut rhizosphere soil microbial community. (A) Changes in relative proportion of the 10 dominant bacteria genera in rhizosphere soil of peanut. (B) Changes in relative proportion of the 10 dominant fungi genera in rhizosphere soil of peanut; Each value represents the mean ± standard error of values from three replicates per treatment. Different letters above error bars indicate significant differences according to Duncan’s test (*P*<0.05).

# 1.3
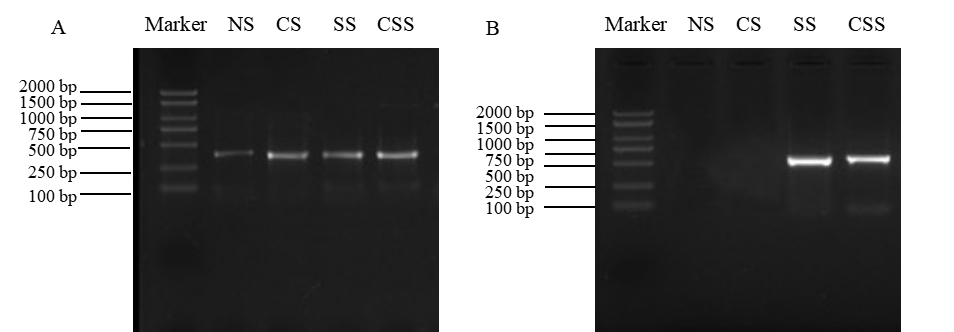


**Supplementary Figure 3.** Electrophoresis representation of *Bacillus* spp. and *Sclerotium rolfsii* DNA presence in peanut rhizosphere soil DNA. (A) Electrophoresis representation of *Bacillus* spp. amplified by B1F/B1R;(B)Electrophoresis representation of *S. rolfsii*. amplified by SRITSF/SRITSR. NS: uninoculated soil; CS: Soil inoculated with *Bacillus velezensis* CB13; SS: Soil inoculated with *Sclerotium rolfsii*; CSS: Soil inoculated with *B. velezensis* CB13 and *S. rolfsii*.

# 2.2 Supplementary Tables

**Supplementary Table 1.** Sequences of primers used in this study

| Gene | Sequence of primer |
| --- | --- |
| 16S rDNA | 27F:5′-AGAGTTTGATCMTGGCTCAG3′  1492R:5′-GGTTACCTTGTTACGACTT3′ |
| *gyrA* | F:5′-CAGTCAGGAAATGCGTACGTCCTT-3′  R: 5′-CAAGGTAATGCTCCAGGCATTGCT-3′ |
| *gyrB* | F: 5′-TTATCTACGACCTTAGACG-3′  R: 5′-TAAATTGAAGTCTTCTCCG-3′ |
| *rpoB* | F: 5′-AGGTCAACTAGT TCAGTATGGAC-3′  R: 5′-AAGAACCA TAACCGGCAACTT-3′ |
| ITS | ITSIF:5′- TGGTCATTTAGAGGAAGTAA-3′  ITS2R:5′-GCTGCGTTCTTCATCGATGC-3′ |
| 16 S V3-V4 | 338F:5′-ACTCCTACGGGAGGCAGCAG-3′  806R:5′-GGACTACHVGGGTWTCTAAT-3′ |
| *Bacillus* spp. | B1F:TTCAAATAGGGCGGCACCTT  B1R:TGCCACCTACGTATTACCGC |
| *Sclerotium rolfsii* | SRITSF:5’-TACACCTGTGAACCAACTG-3’  SRITSR:5’-CATACAAGCTAGAATCCC-3’ |

**Supplementary Table 2.** Physiological and biochemical characteristics of the CB13 strain

| Test item | Result | Test item | Result |
| --- | --- | --- | --- |
| Gram staining | + | Methyl red | - |
| Starch hydrolysis | + | Peptone hydrolysis | - |
| Gelatin liquefication test | + | 3%NaCl | + |
| Oxidase | + | 5%NaCl | + |
| VP test | + | 7% NaCl | + |
| Catalase | + | 9%NaCl | + |
| Nitrate reduction | + | 11%NaCl | + |

+: positive (growth or reaction); –: negative (no growth or no reaction).

**Supplementary Table 3.** Main structure of bacteria communities of soil

| Sample | Reads | Phylum | Class | Order | Family | Genus | OTUs |
| --- | --- | --- | --- | --- | --- | --- | --- |
| NS_1 | 36259 | 31 | 87 | 195 | 310 | 567 | 1821 |
| NS_2 | 37047 | 29 | 80 | 199 | 308 | 565 | 1782 |
| NS_3 | 37446 | 33 | 88 | 202 | 316 | 575 | 1843 |
| CS_1 | 29511 | 35 | 105 | 238 | 373 | 665 | 2045 |
| CS_2 | 26649 | 34 | 102 | 230 | 357 | 648 | 1997 |
| CS_3 | 32167 | 35 | 107 | 250 | 385 | 698 | 2195 |
| SS_1 | 34895 | 31 | 79 | 190 | 309 | 567 | 1695 |
| SS_2 | 44340 | 29 | 84 | 196 | 310 | 578 | 1733 |
| SS_3 | 39513 | 29 | 88 | 205 | 324 | 598 | 1910 |
| CSS_1 | 34304 | 29 | 84 | 209 | 317 | 577 | 1833 |
| CSS_2 | 36936 | 30 | 81 | 193 | 311 | 585 | 1841 |
| CSS_3 | 31256 | 33 | 84 | 196 | 307 | 554 | 1731 |

NS: uninoculated soil; CS: Soil inoculated with *Bacillus velezensis* CB13; SS: Soil inoculated with *Sclerotium rolfsii*; CSS: Soil inoculated with *B. velezensis* CB13 and *S. rolfsii*.

**Supplementary Table 4.** Main structure of Fungi communities of soil

| Sample | Reads | Phylum | Class | Order | Family | Genus | OTUs |
| --- | --- | --- | --- | --- | --- | --- | --- |
| NS_1 | 76360 | 10 | 29 | 65 | 125 | 197 | 531 |
| NS_2 | 72080 | 10 | 29 | 63 | 123 | 196 | 498 |
| NS_3 | 64377 | 11 | 29 | 57 | 113 | 181 | 481 |
| CS_1 | 57472 | 9 | 24 | 59 | 109 | 178 | 453 |
| CS_2 | 83454 | 11 | 32 | 66 | 114 | 182 | 500 |
| CS_3 | 59973 | 9 | 25 | 56 | 105 | 163 | 433 |
| SS_1 | 63004 | 9 | 25 | 59 | 114 | 182 | 447 |
| SS_2 | 63461 | 9 | 26 | 58 | 108 | 171 | 421 |
| SS_3 | 65629 | 7 | 24 | 55 | 106 | 177 | 405 |
| CSS_1 | 63779 | 10 | 25 | 60 | 108 | 175 | 474 |
| CSS_2 | 58757 | 9 | 30 | 63 | 107 | 168 | 472 |
| CSS_3 | 57323 | 10 | 32 | 69 | 122 | 197 | 509 |

NS: uninoculated soil; CS: Soil inoculated with *Bacillus velezensis* CB13; SS: Soil inoculated with *Sclerotium rolfsii*; CSS: Soil inoculated with *B. velezensis* CB13 and *S. rolfsii*.

**Supplementary Table 5.** Composition and relative abundance of predominant bacterial phylum in peanut rhizosphere soil

| Bacteria | NS | CS | SS | CSS |
| --- | --- | --- | --- | --- |
| *Actinobacteriota* | 49.86±0.43b | 33.21±0.31a | 50.11±1.43b | 38.67±0.82a |
| *Proteobacteria* | 21.05±0.73a | 32.06±1.12c | 25.48±3.04b | 28.70±1.75b |
| *Chloroflexi* | 11.36±0.24c | 8.71±0.85ab | 8.40±1.83a | 9.90±1.01b |
| *Acidobacteriota* | 3.47±0.18b | 5.95±0.64d | 2.83±0.47a | 4.73±0.34c |
| *Bacteroidota* | 3.55±0.22a | 4.31±0.19a | 4.08±0.38a | 4.63±0.40a |
| *Firmicutes* | 3.09±0.36b | 4.62±0.42c | 2.36±0.05a | 3.64±0.06b |
| *Patescibacteria* | 3.21±0.12ab | 3.22±0.16b | 2.85±0.15a | 3.85±0.23ab |
| *Gemmatimonadota* | 2.01±0.04a | 4.00±0.26c | 1.94±0.23a | 2.99±0.11b |
| *Myxococcota* | 0.39±0.02a | 1.42±0.07c | 0.37±0.04a | 0.81±0.03b |
| Others | 2.01±0.12b | 2.5±0.22c | 1.58±0.23a | 2.09±0.19b |

NS: uninoculated soil; CS: Soil inoculated with *Bacillus velezensis* CB13; SS: Soil inoculated with *Sclerotium rolfsii*; CSS: Soil inoculated with *B. velezensis* CB13 and *S. rolfsii*.

**Supplementary Table 6.** Composition and relative abundance of predominant fungal phylum in peanut rhizosphere soil

| Bacteria | NS | CS | SS | CSS |
| --- | --- | --- | --- | --- |
| *Ascomycota* | 89.39±1.26b | 91.41±0.41b | 58.77±1.23a | 88.28±0.94b |
| *Basidiomycota* | 2.78±0.62a | 2.85±0.23a | 37.26±1.17b | 3.30±0.64a |
| Unclassified*_*k*_Fungi* | 2.99±0.30a | 2.57±0.17a | 2.64±0.10a | 6.01±0.66b |
| *Mortierellomycota* | 2.03±0.23ab | 2.41±0.33b | 1.06±0.30a | 1.66±0.33ab |
| *Olpidiomycota* | 1.78±0.25b | 0.21±0.04a | 0.00±0a | 0.40±0.18ab |
| Others | 0.85±0.26b | 0.55±0.09ab | 0.26±0.04a | 0.36±0.05a |

Data are presented as *x±s*(*n*=3). Means followed by a different letter in the columns indicate significant differences according to one-way analysis of variance (*P*<0.05). NS: uninoculated soil; CS: Soil inoculated with *Bacillus velezensis* CB13; SS: Soil inoculated with *Sclerotium rolfsii*; CSS: Soil inoculated with *B. velezensis* CB13 and *S. rolfsii*.

**Supplementary Table 7.** Differential abundance analysis of predominant bacterial and fungal phylum in peanut rhizosphere soil

| Bacteria | NS_mean | CS_mean | log2(fc) | PValue | FDR |
| --- | --- | --- | --- | --- | --- |
| Myxococcota | 3867.25 | 15671.58 | 2.02 | 0.00 | 0.01 |
| Gemmatimonadota | 20098.64 | 42541.56 | 1.08 | 0.01 | 0.05 |
| Proteobacteria | 36.48 | 800.83 | 4.46 | 0.02 | 0.08 |
| Firmicutes | 1011.23 | 4158.38 | 2.04 | 0.03 | 0.12 |
| Actinobacteriota | 498582.62 | 315532.81 | -0.66 | 0.03 | 0.12 |
| Fungi | NS_mean | CS_mean | log2(fc) | PValue | FDR |
| Olpidiomycota | 17804.19 | 2121.26 | -3.07 | 0.00 | 0.01 |

**Supplementary Table 8.** Composition and relative abundance of bacterial function in peanut rhizosphere soil according to the Kyoto Encyclopedia of Genes and Genomes (KEGG) pathway (Secondary functional layer, abundance ratio higher than 0.1%)

| Bacterial function | NS | CS | SS | CSS |
| --- | --- | --- | --- | --- |
| Global and overview maps | 40.55±0.02a | 40.55±0.11a | 40.83±0.07b | 40.53±0.13a |
| Carbohydrate metabolism | 9.70±0.05a | 9.70±0.12a | 9.98±0.08b | 9.65±0.14a |
| Amino acid metabolism | 8.40±0.02a | 8.44±0.02a | 8.50±0.01b | 8.42±0.02a |
| Energy metabolism | 4.30±0.01a | 4.31±0.02a | 4.29±0.01a | 4.32±0.01a |
| Metabolism of cofactors and vitamins | 4.07±0.01b | 4.05±0.01b | 4.03±0.00a | 4.06±0.01b |
| Membrane transport | 2.92±0.01a | 2.95±0.02a | 2.92±0.01a | 2.95±0.01a |
| Lipid metabolism | 2.42±0.01a | 2.44±0.01a | 2.43±0.01a | 2.43±0.01a |
| Translation | 2.39±0.02b | 2.36±0.02ab | 2.34±0.00a | 2.37±0.01ab |
| Xenobiotics biodegradation and metabolism | 2.30±0.02a | 2.35±0.04a | 2.32±0.02a | 2.33±0.03a |
| Signal transduction | 2.31±0.01b | 2.29±0.02ab | 2.24±0.01a | 2.30±0.03b |
| Replication and repair | 2.27±0.01a | 2.26±0.01a | 2.26±0.00a | 2.26±0.01a |
| Cellular community - prokaryotes | 2.23±0.01ab | 2.25±0.02ab | 2.20± 0.01a | 2.26±0.02b |
| Nucleotide metabolism | 2.17±0.01a | 2.16±0.01a | 2.16±0.00a | 2.16±0.01a |
| Metabolism of other amino acids | 1.63±0.00b | 1.63±0.00b | 1.60±0.00a | 1.63±0.01b |
| Biosynthesis of other secondary metabolites | 1.58±0.00a | 1.57±0.00a | 1.57±0.00a | 1.57±0.01a |
| Folding, sorting and degradation | 1.24±0.01a | 1.23±0.01a | 1.23±0.00a | 1.23±0.01a |
| Metabolism of terpenoids and polyketides | 1.19±0.01a | 1.20±0.01a | 1.20±0.01a | 1.20±0.02a |
| Glycan biosynthesis and metabolism | 1.13±0.01b | 1.11±0.00b | 1.09±0.01a | 1.11±0.01b |
| Cell growth and death | 0.82±0.01b | 0.81±0.01b | 0.77±0.01a | 0.82±0.02b |
| Endocrine system | 0.76±0.00a | 0.77±0.00b | 0.78±0.00b | 0.77±0.01b |
| Drug resistance: antimicrobial | 0.65±0.01b | 0.64±0.01b | 0.61±0.01a | 0.64±0.01b |
| Cell motility | 0.64±0.01b | 0.63±0.03b | 0.56±0.01a | 0.65±0.03b |
| Infectious disease: bacterial | 0.60±0.00b | 0.59±0.01b | 0.56±0.01a | 0.59±0.01b |
| Cancer: overview | 0.55±0.00a | 0.55±0.00a | 0.54±0.01a | 0.55±0.01a |
| Aging | 0.45±0.00b | 0.45±0.01b | 0.43±0.01a | 0.45±0.01b |
| Transport and catabolism | 0.34±0.00a | 0.34±0.00a | 0.34±0.00a | 0.34±0.01a |
| Neurodegenerative disease | 0.32±0.00b | 0.32±0.01b | 0.28±0.01a | 0.32±0.01b |
| Environmental adaptation | 0.26±0.00b | 0.26±0.00b | 0.25±0.00a | 0.27±0.01b |
| Drug resistance: antineoplastic | 0.25±0.00b | 0.25±0.00b | 0.24±0.00a | 0.25±0.01b |
| Endocrine and metabolic disease | 0.24±0.00a | 0.23±0.00a | 0.23±0.00a | 0.23±0.00a |
| Infectious disease: viral | 0.23±0.00b | 0.22±0.01b | 0.19±0.01a | 0.23±0.01b |
| Nervous system | 0.21±0.00a | 0.21±0.00a | 0.21±0.00a | 0.21±0.00a |
| Cardiovascular disease | 0.19±0.00b | 0.19±0.00b | 0.17±0.00a | 0.19±0.01b |
| Cancer: specific types | 0.17±0.00b | 0.17±0.01b | 0.15±0.01a | 0.17±0.01b |
| Immune system | 0.13±0.00a | 0.12±0.00a | 0.12±0.00a | 0.12±0.00a |
| Transcription | 0.12±0.00a | 0.12±0.00a | 0.12±0.00a | 0.12±0.00a |

Data are presented as *x±s* (*n*=3). Means followed by a different letter in the columns indicate significant differences according to one-way analysis of variance (*P*<0.05). NS: uninoculated soil; CS: Soil inoculated with *Bacillus velezensis* CB13; SS: Soil inoculated with *Sclerotium rolfsii*; CSS: Soil inoculated with *B. velezensis* CB13 and *S. rolfsii*.

**Supplementary Table 9.** Composition and relative abundance of fungi function in peanut rhizosphere soil according to the MetaCyc pathwayr (abundance ratio higher than 0.1%)

| Fungal function | NS | CS | SS | CSS |
| --- | --- | --- | --- | --- |
| aerobic respiration I (cytochrome c) | 6.17±0.17a | 6.18±0.17a | 6.16±0.16a | 6.28±0.02a |
| aerobic respiration II (cytochrome c) (yeast) | 6.17±0.17a | 6.18±0.17a | 6.16±0.16a | 6.28±0.02a |
| glyoxylate cycle | 2.80±0.01a | 2.82±0.02ab | 2.90±0.01c | 2.84±0.01b |
| pentose phosphate pathway (non-oxidative branch) | 2.74±0.05b | 2.79±0.08b | 2.53±0.04a | 2.76±0.03b |
| fatty acid &beta;-oxidation (peroxisome, yeast) | 2.47±0.13a | 2.38±0.03a | 3.28±0.06b | 2.41±0.06a |
| guanosine nucleotides degradation II | 2.38±0.01a | 2.30±0.06a | 2.31±0.09a | 2.38±0.02a |
| D-myo-inositol (1,4,5)-trisphosphate biosynthesis | 2.32±0.02b | 2.30±0.02b | 2.13±0.03a | 2.28±0.03b |
| palmitate biosynthesis I (animals and fungi) | 2.34±0.18b | 2.44±0.10b | 1.80±0.04a | 2.34±0.03b |
| TCA cycle II (plants and fungi) | 2.21±0.01a | 2.22±0.02a | 2.26±0.01b | 2.23±0.00ab |
| adenosine ribonucleotides de novo biosynthesis | 2.13±0.02a | 2.15±0.02a | 2.17±0.01a | 2.16±0.01a |
| pyruvate fermentation to isobutanol (engineered) | 2.14±0.01a | 2.15±0.02a | 2.16±0.01a | 2.16±0.00a |
| tRNA charging | 2.11±0.00b | 2.13±0.03b | 2.06±0.02a | 2.13±0.00b |
| GDP-mannose biosynthesis | 2.12±0.02b | 2.04±0.02a | 2.07±0.01ab | 2.06±0.02a |
| superpathway of adenosine nucleotides de novo biosynthesis I | 2.01±0.02a | 2.03±0.03a | 2.08±0.01a | 2.04±0.01a |
| L-valine biosynthesis | 1.98±0.01a | 1.99±0.02a | 1.96±0.01a | 2.00±0.00a |
| superpathway of adenosine nucleotides de novo biosynthesis II | 1.92±0.03a | 1.91±0.02a | 1.95±0.01a | 1.93±0.01a |
| superpathway of L-serine and glycine biosynthesis I | 1.91±0.01b | 1.92±0.03b | 1.84±0.01a | 1.92±0.01b |
| methyl ketone biosynthesis | 1.95±0.08b | 1.87±0.01ab | 1.79±0.06a | 1.89±0.03ab |
| glycogen biosynthesis II (from UDP-D-Glucose) | 1.85±0.02a | 1.87±0.04a | 1.91±0.02a | 1.86±0.01a |
| pentose phosphate pathway | 1.86±0.03ab | 1.89±0.04b | 1.81±0.03a | 1.88±0.00ab |
| phosphopantothenate biosynthesis I | 1.80±0.02ab | 1.83±0.03b | 1.76±0.02a | 1.82±0.00b |
| fatty acid beta-oxidation V (unsaturated, odd number, di-isomerase-dependent) | 1.79±0.09a | 1.78±0.05a | 1.80±0.09a | 1.79±0.04a |
| NAD/NADH phosphorylation and dephosphorylation | 1.73±0.01a | 1.79±0.03b | 1.77±0.03ab | 1.81±0.01b |
| octanoyl-[acyl-carrier protein] biosynthesis (mitochondria, yeast) | 1.89±0.04b | 1.93±0.01b | 1.31±0.05a | 1.88±0.02b |
| D-galactose degradation V (Leloir pathway) | 1.74±0.01a | 1.77±0.03a | 1.72±0.02a | 1.78±0.01a |
| superpathway of phosphatidate biosynthesis (yeast) | 1.70±0.05a | 1.73±0.05a | 1.70±0.04a | 1.74±0.00a |
| 4-amino-2-methyl-5-phosphomethylpyrimidine biosynthesis (yeast) | 1.66±0.00a | 1.69±0.02b | 1.73±0.01c | 1.69±0.00b |
| pyrimidine deoxyribonucleotides de novo biosynthesis I | 1.62±0.04ab | 1.59±0.01a | 1.66±0.01b | 1.61±0.01ab |
| galactose degradation I (Leloir pathway) | 1.64±0.01b | 1.69±0.04b | 1.38±0.04a | 1.66±0.02b |
| superpathway of guanosine nucleotides de novo biosynthesis I | 1.59±0.03a | 1.57±0.01a | 1.58±0.00a | 1.59±0.01a |
| superpathway of pyrimidine nucleobases salvage | 1.59±0.03a | 1.57±0.02a | 1.58±0.01a | 1.59±0.01a |
| superpathway of L-threonine biosynthesis | 1.53±0.02a | 1.56±0.02a | 1.56±0.01a | 1.56±0.00a |
| L-proline biosynthesis II (from arginine) | 1.52±0.01a | 1.53±0.02a | 1.51±0.01a | 1.54±0.01a |
| formaldehyde assimilation III (dihydroxyacetone cycle) | 1.52±0.01b | 1.52±0.00b | 1.49±0.01a | 1.52±0.01b |
| chitin degradation to ethanol | 1.61±0.04b | 1.51±0.06ab | 1.44±0.02a | 1.47±0.09ab |
| glycolysis III (from glucose) | 1.50±0.02a | 1.52±0.02a | 1.49±0.02a | 1.52±0.01a |
| urea cycle | 1.48±0.00a | 1.50±0.02a | 1.48±0.01a | 1.50±0.01a |
| pyrimidine deoxyribonucleotides biosynthesis from CTP | 1.53±0.05b | 1.50±0.03b | 1.32±0.04a | 1.51±0.01b |
| CDP-diacylglycerol biosynthesis I | 1.45±0.04a | 1.47±0.05a | 1.44±0.04a | 1.48±0.01a |
| L-leucine degradation I | 1.50±0.05b | 1.59±0.08b | 1.17±0.10a | 1.58±0.02b |
| heme biosynthesis I (aerobic) | 1.41±0.00a | 1.42±0.01a | 1.58±0.02b | 1.42±0.00a |
| guanosine ribonucleotides de novo biosynthesis | 1.44±0.01a | 1.46±0.02a | 1.45±0.01a | 1.46±0.01a |
| mevalonate pathway I | 1.46±0.02a | 1.44±0.01a | 1.44±0.00a | 1.46±0.01a |
| superpathway of heme biosynthesis from glycine | 1.39±0.02a | 1.42±0.01a | 1.49±0.01b | 1.41±0.00a |
| tetrapyrrole biosynthesis II (from glycine) | 1.38±0.03a | 1.42±0.01ab | 1.43±0.01b | 1.41±0.00ab |
| pyrimidine deoxyribonucleotide phosphorylation | 1.42±0.04a | 1.39±0.02a | 1.39±0.01a | 1.40±0.01a |
| UDP-N-acetyl-D-glucosamine biosynthesis II | 1.36±0.01a | 1.36±0.02a | 1.37±0.01a | 1.37±0.01a |
| 1,3-propanediol biosynthesis (engineered) | 1.40±0.01b | 1.43±0.03b | 1.20±0.02a | 1.42±0.01b |
| sulfate reduction I (assimilatory) | 0.33±0.11a | 0.38±0.11a | 0.90±0.07b | 0.38±0.02a |
| L-tyrosine degradation I | 0.55±0.05b | 0.47±0.11ab | 0.35±0.08a | 0.46±0.03ab |
| gluconeogenesis I | 0.44±0.04a | 0.45±0.09a | 0.32±0.04a | 0.48±0.06a |
| adenine and adenosine salvage III | 0.38±0.02b | 0.23±0.03a | 0.76±0.03c | 0.28±0.04a |
| octane oxidation | 0.19±0.07a | 0.14±0.03a | 1.01±0.05b | 0.22±0.03a |
| sucrose degradation III (sucrose invertase) | 0.32±0.17a | 0.38±0.14a | 0.32±0.11a | 0.29±0.01a |
| L-tryptophan degradation to 2-amino-3-carboxymuconate semialdehyde | 0.15±0.10a | 0.17±0.08a | 0.65±0.07b | 0.12±0.01a |
| stearate biosynthesis III (fungi) | 0.26±0.15a | 0.31±0.11a | 0.27±0.11a | 0.23±0.01a |
| NAD/NADP-NADH/NADPH mitochondrial interconversion (yeast) | 0.25±0.15a | 0.30±0.12a | 0.26±0.11a | 0.21±0.01a |
| NAD/NADP-NADH/NADPH cytosolic interconversion (yeast) | 0.18±0.11a | 0.22±0.09a | 0.19±0.08a | 0.16±0.01a |
| superpathway of ubiquinol-6 biosynthesis (eukaryotic) | 0.11±0.02a | 0.13±0.03a | 0.21±0.07a | 0.14±0.00a |
| superpathway of purine nucleotide salvage | 0.14±0.08a | 0.17±0.07a | 0.15±0.06a | 0.12±0.01a |
| phospholipid remodeling (phosphatidylethanolamine, yeast) | 0.28±0.11c | 0.09±0.02ab | 0.05±0.01a | 0.13±0.03ab |
| trehalose degradation V | 0.13±0.08a | 0.16±0.06a | 0.14±0.06a | 0.11±0.01a |
| glucose and glucose-1-phosphate degradation | 0.13±0.08a | 0.15±0.06a | 0.13±0.06a | 0.11±0.01a |
| monoacylglycerol metabolism (yeast) | 0.13±0.08a | 0.15±0.06a | 0.13±0.06a | 0.11±0.01a |
| phospholipases | 0.20±0.05b | 0.07±0.01a | 0.06±0.01a | 0.09±0.01a |
| 5-aminoimidazole ribonucleotide biosynthesis I | 0.09±0.03a | 0.10±0.02a | 0.11±0.01a | 0.10±0.01a |

Data are presented as *x±s* (*n*=3). Means followed by a different letter in the columns indicate significant differences according to one-way analysis of variance (*P*<0.05). NS: uninoculated soil; CS: Soil inoculated with *Bacillus velezensis* CB13; SS: Soil inoculated with *Sclerotium rolfsii*; CSS: Soil inoculated with *B. velezensis* CB13 and *S. rolfsii*.
